# Hybrid Androgen Receptor Inhibitors Outperform Enzalutamide and EPI‐001 in *in vitro* Models of Prostate Cancer Drug Resistance

**DOI:** 10.1002/cmdc.202200548

**Published:** 2022-11-15

**Authors:** Radu Costin Bizga Nicolescu, Zoe R. Maylin, Francisco Javier Pérez‐Areales, Jessica Iegre, Hardev S. Pandha, Mohammad Asim, David R. Spring

**Affiliations:** ^1^ Yusuf Hamied Department of Chemistry University of Cambridge Lensfield Road Cambridge CB2 1EW UK; ^2^ Department of Clinical and Experimental Medicine University of Surrey Guildford GU2 TXH UK

**Keywords:** androgen receptor, dual inhibitors, prostate cancer, enzalutamide, EPI-001.

## Abstract

Androgen receptor targeted therapies for prostate cancer have serious limitations in advanced stages of the disease. While resistance to the FDA‐approved enzalutamide is extensively documented, novel therapies based on epichlorohydrin scaffolds (EPI) are currently in clinical trials, but display suboptimal pharmacokinetics. Herein, we report the synthesis and biological characterisation of a novel class of compounds designed through covalently linking enzalutamide and EPI‐001 through various triazole based linkers. The compounds display an 18 to 53 fold improvement in the cell killing potency towards C4‐2b prostate cancer (PCa) cells compared to the gold standards of therapy, enzalutamide and EPI‐001. The most promising compounds were proven to exhibit their toxicity exclusively through androgen receptor (AR) mediated pathways. This work sets the basis for the first class of hybrid AR inhibitors which successfully combine two drug moieties – EPI‐001 and enzalutamide – into the same molecule.

## Graphical abstract

**Combining two drugs in one**? A novel series of androgen receptor (AR) targeting compounds for prostate cancer use a novel, exciting strategy – covalently linking Enzalutamide and EPI‐001. Significant improvement in cell killing potencies were observed and proven to be AR mediated.

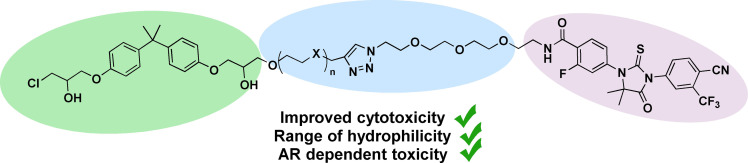

## Introduction

The androgen receptor (AR) is a hormone‐activated transcription factor and is the main driver of PCa. AR activity in healthy, non‐cancerous epithelial cells promotes the development and maintenance of the male reproductive system and has a wider role in other biological processes. However, dysregulation of this signalling can result in the activation of oncogenic transcription programmes that can trigger uncontrolled proliferation of cells, and thus transformation to an aggressive phenotype culminating in tumour formation.^[1]^

While PCa patients with localised disease are treated with focal therapies or radical prostatectomy, treatment of metastatic disease requires androgen‐deprivation therapy (ADT). Despite a high rate of initial response, almost all patients progress to a more advanced and incurable disease known as Castration‐Resistant Prostate Cancer (CRPC). It is well known that AR signalling is maintained or restored in CRPC despite low serum levels of circulating androgens achieved by ADT.^[2]^ Patients will then receive second‐generation AR inhibitors such as enzalutamide which competitively binds to the AR ligand‐binding domain (LBD), outcompeting dihydrotestosterone and thus inhibiting downstream AR signalling and causing PCa cancer to regress.

Enzalutamide is an FDA‐approved non‐steroidal antiandrogen for metastatic CRPC, which is administered together with ADT as well as monotherapy for CRPC.^[3]^ A crucial limitation of the treatment is the development of resistance, with AR signalling becoming unresponsive to enzalutamide.^[4]^ Apart from enzalutamide, a variety of steroidal and non‐steroidal anti androgens have been developed to date.^[5]^ Notably, resistance to enzalutamide arises primarily due to AR variants (AR‐Vs) which lack the LBD and are able to sustain AR signalling in the presence of enzalutamide.^[6]^ Overall, these limitations highlight a need for complementary strategies to inhibit the AR.

The priority as an alternative to LBD inhibition has been inhibiting the N‐terminal domain (NTD). This campaign has yielded a promising class of bisphenol A derived compounds, under the generic name of EPI analogues. Two EPI derivatives with undisclosed structures are currently in clinical trials for CRPC, highlighting their therapeutic potential.^[7]^ The compound EPI‐001 represents a mixture of four stereoisomers which are able to inhibit protein‐protein interactions between AR‐NTD and RAP74,^[8]^ an essential component of the AR transcriptional machinery. More specifically, EPI‐001 binds to a region in the AR‐NTD called transactivation‐unit 5 on the AR NTD, corresponding to residues 361‐537.^[9]^ Because EPIs bind to the AR‐NTD, they effectively inhibit a broad range of AR‐Vs, most of which are implicated in the development of CRPC.^[10]^ Notably, EPI inhibitors inhibit constitutively active, hormone independent AR variants that lack their LBD, as well as AR variants which have acquired gain‐of‐function mutations in the LBD.^[11]^ Even if EPIs are efficient at inhibiting CRPC specific splice variants that lack their LBD, they display poor pharmacokinetics properties and half inhibitory concentrations (IC_50_) in the high micromolar range.[^12^, ^13^, ^14^] Hence, patients suffer from excessive pill burden. It has been shown that patients have significantly lower circulating doses than what would be required *in vitro* for optimal therapeutic concentrations.^[15]^ Hence, structural modifications are needed in order to improve EPI's inhibition profile.

**Figure 1:**
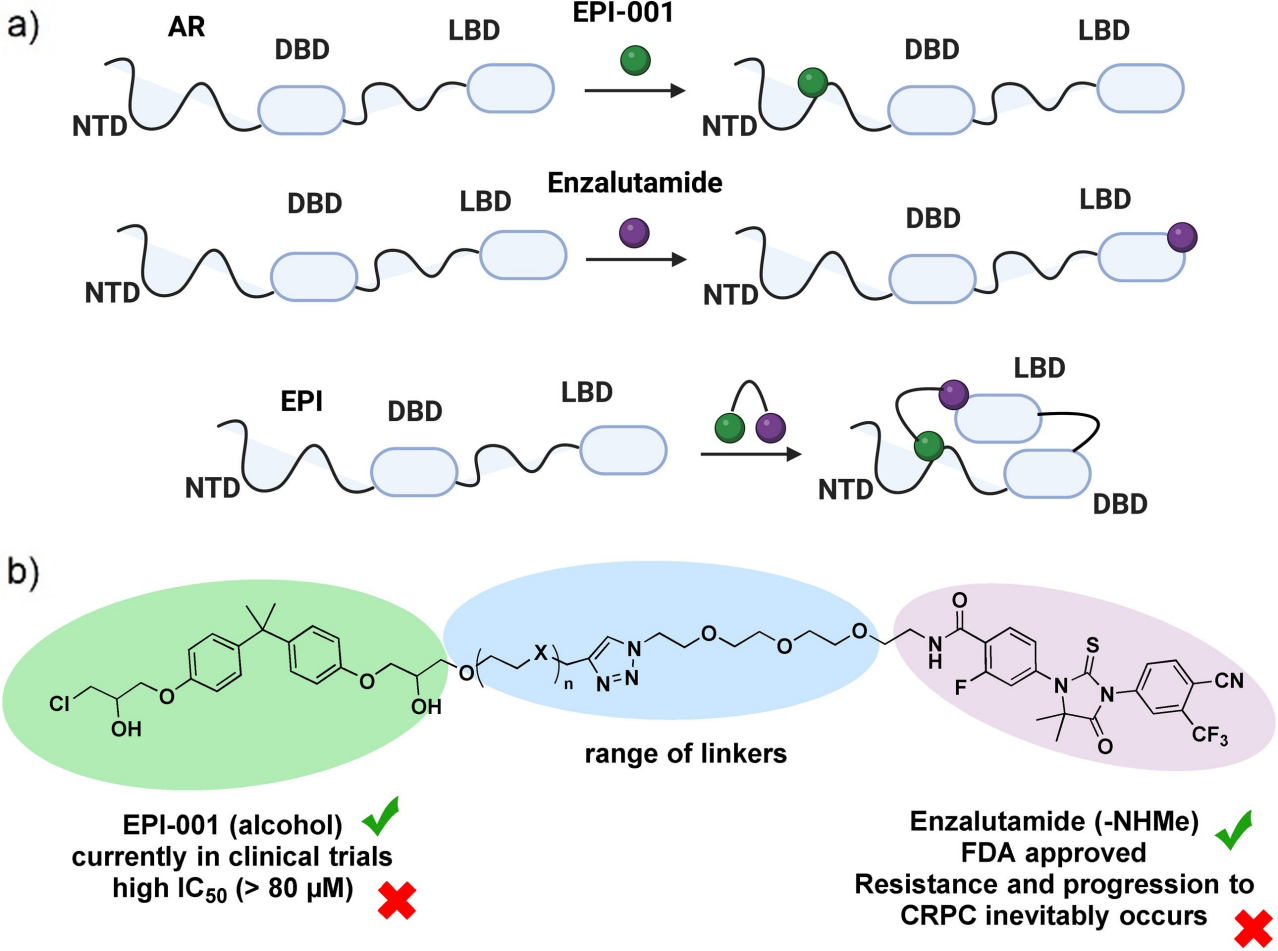
a) Targeted design of dual inhibitors and their mechanism of action. AR=androgen receptor; NTD=N‐terminal domain; DBD=DNA binding domain; LBD=ligand binding domain. b) Several limitations of Enzalutamide and EPI which could be overcome by the introduction of linkers. Structures for EPI‐001 and enzalutamide are shown in Figure 2. Figure 1a created with BioRender.

This work explores the *in vitro* potential of new hybrid compounds that simultaneously target two sites of the AR, the N‐terminal domain (NTD) and the ligand binding domain (LBD) (Figure 1). A dual target strategy by covalently linking enzalutamide to another drug has been recently successfully implemented for the development of dual inhibitors between enzalutamide and etinostat, a histone deacetylase inhibitor.^[16]^ Similarly, there are extensive precedents for the success of heterobifunctional molecules, such as PROTACs,^[17]^ bifunctional therapeutics^[18]^ and multitarget compounds.^[19]^ Moreover, significant efforts in moving away from conventional active site targeting have been attempted up to date.[^20^, ^21^] As such, five compounds which covalently link enzalutamide and EPI‐001 with different linker lengths were synthesised in order to explore the potential dual domain inhibition. Various triazole‐PEG linkers were chosen, aiming to cover a range of different linker lengths. Triazole linkers are known to be biocompatible and have been widely used.[^22^, ^23^] This strategy could also improve binding affinity through an entropy driven effect. Furthermore, covalently linking two pharmacophores could help overcome resistance to conventional enzalutamide only based therapy.

## Results and Discussion

### Synthetic strategy

**Figure 2:**
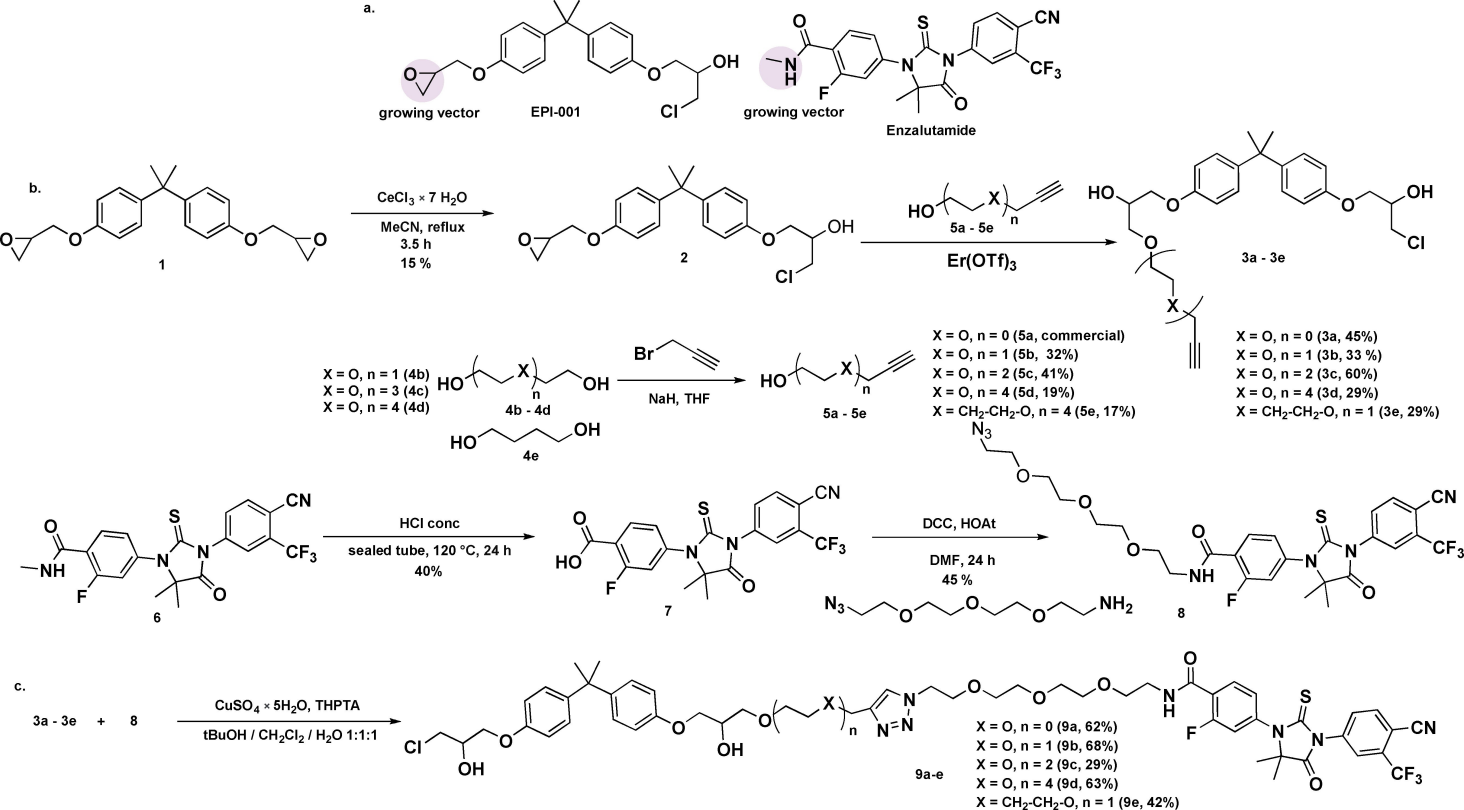
a) Structures of EPI‐001 and enzalutamide, with highlighted growing vectors for linker attachment. b) Synthetic route towards the dual inhibitors. c) Highlighted structures of the hybrid inhibitors.

Five compounds were synthesised exploring various linker types and sizes between an enzalutamide and an EPI‐001 moiety. The strategy aimed at linking enzalutamide through a previously explored growing vector to the EPI moiety.^[24]^ The EPI moiety was functionalised in a way that preserved its essential chloride moiety,^[25]^ while the opposite end was used as a growing vector for linker attachment. The compounds were synthesised as detailed in Figure 2b. A family of alkyne‐alcohols **5 a**–**5 e** was reacted with the mono‐chloro derivative **2** in an erbium (III) catalysed epoxide ring opening to yield **3 a**–**3 e**. A difficult hydrolysis of enzalutamide **6** yielded carboxylic acid **7** which was coupled to an azido‐containing linker to yield **8**. Copper catalysed azide‐alkyne cycloadditions were chosen as the last step to yield the final compounds **9 a**–**9 e**.

### Biological characterisation

#### Cellular toxicity assays

The five compounds were tested against a C4‐2b prostate cancer cell line. The C4‐2b cell line is sequentially derived from the androgen independent C4‐2 and androgen sensitive LNCaP cell lines that originated from a patient with metastatic carcinoma. C4‐2b are a more aggressive sub‐line of these cells. Unlike LNCaP, C4‐2b expresses several AR‐Vs (Supplementary Figure 1), this displaying enzalutamide resistance, making it a promising testing system for our compounds.[^26^, ^27^]

**Figure 3:**
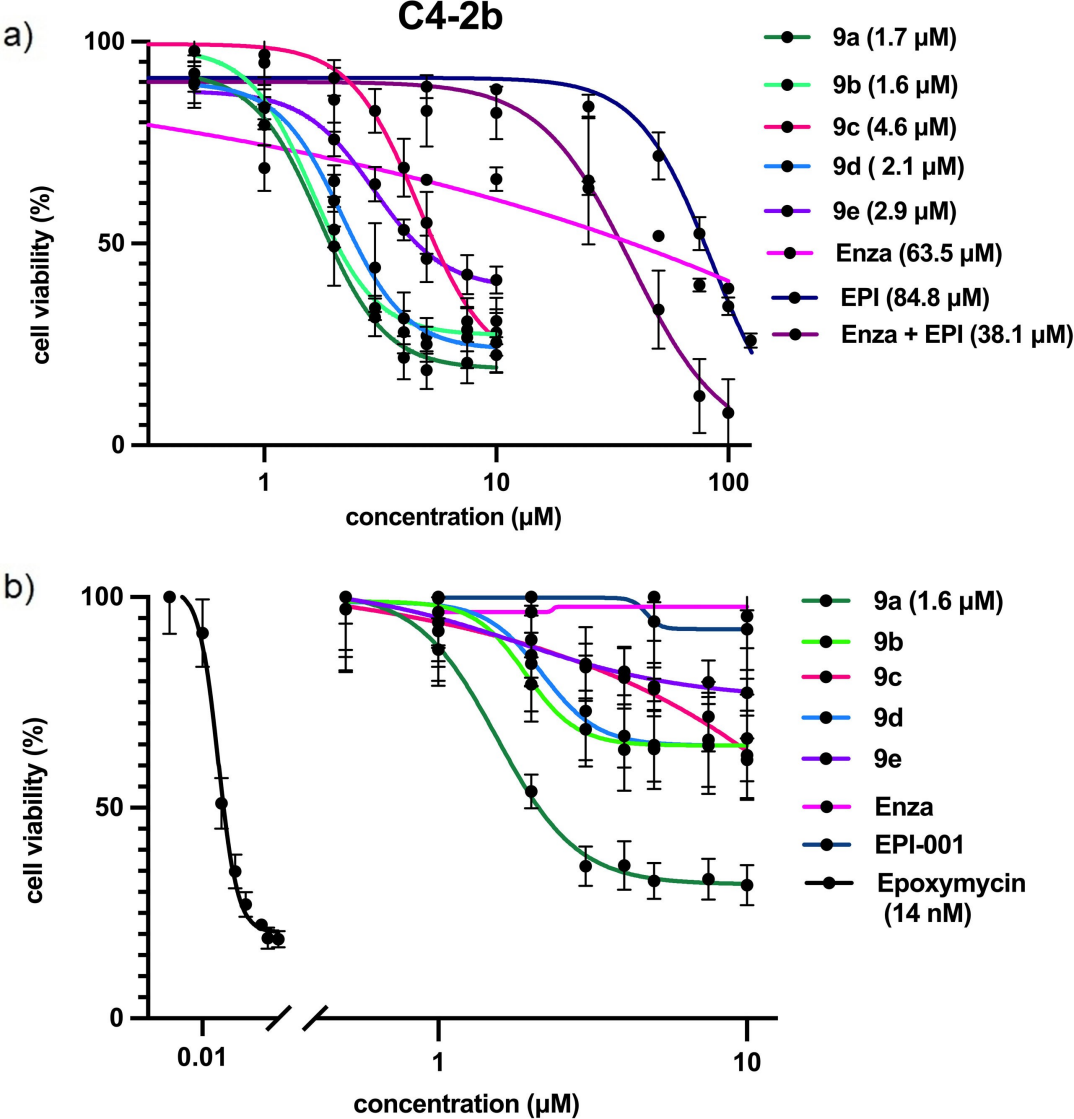
a) Cell toxicity assays recorded with **9 a**–**9 e** in C4‐2b cells. b) Cell toxicity assays recorded with **9 a**–**9 e** in PC3 cells. Experiments performed in 3 biological replicates and 3 technical replicates.

Cellular toxicity studies showed that compounds **9 a**–**9 e** display an 18 to 53 fold improvement in the half lethal concentration (LC_50_) compared to enzalutamide and EPI‐001 against the C4‐2b prostate cancer cell line (LC_50_s ranging between 1.7 and 4.6 μM vs. 63.5 μM for enzalutamide and 84.8 μM for EPI‐001) (Figure 3a). Enzalutamide displayed a non‐sigmoidal response over the interval of tested concentrations, in accordance to previous findings^,[28]^.^[29]^ EPI was found to have an LC_50_ of 84.4 μM, in accordance with literature estimates.^[14]^ An equimolar cocktail of enzalutamide and EPI‐001 outperformed both EPI‐001 and enzalutamide (LC_50_=38.1 μM), presumably due to the genetic heterogeneity of the C4‐2b cell line and prevalence of variants (Supporting Information Figure 1).^[30]^ Crucially, compounds **9 a**–**9 e** outperform both enzalutamide and EPI‐001 by factors of 18 to 53, highlighting the success of the hybridisation strategy.

To evaluate whether the cell toxicity was AR mediated, we performed complementary cytotoxicity studies on PC‐3 cells. PC‐3 cells are metastatic adenocarcinoma cells that express very low levels of AR^[31]^ and therefore both enzalutamide and EPI‐001 are not toxic at concentrations lower than 100 μM.^[32]^ Compounds **9 a**–**9 e** were therefore expected to not display any significant toxicity towards the PC‐3 cell line.

The results presented in Figure 3 show that 9e has non‐significant toxicity towards the PC‐3 cells, while 9a has significant non‐AR mediated toxicity with an LC_50_ of 1.6 μM. It is therefore likely that **9a**’s performance in C4‐2b cells is non‐AR mediated given its identical LC_50_ values in these two cells lines (1.6 μM and 1.7 μM respectively). Notably, compounds **9 b**–**9 d** displayed negligible toxicity towards the PC‐3 cell line, suggesting that their toxicity is AR dependent (Figure 3b).

#### Luciferase reporter assays

Aiming to prove the direct interaction of the dual inhibitors with the AR, target engagement luciferase assays were performed. AR‐null CV‐1 monkey kidney cells were transfected with a Gal_4_‐AR‐FL plasmid construct that can be induced by androgens and can interact with Gal_4_‐DNA binding sites present on the co‐transfected reporter construct. This highly sensitive system reports directly on the AR‐ mediated transcriptional activity. In this assay, synthetic androgen metribolone (R1881) treatment (1 nM) led to a four‐fold increase in luciferase activity, indicating androgen mediated AR activation, as expected. Co‐treatment with enzalutamide at a concentration of 5 μM led to a significant decrease in luciferase activity, while EPI‐001 was not able to inhibit gene transcription at the tested concentration (5 μM), in accordance to previous findings.^[32]^

**Figure 4:**
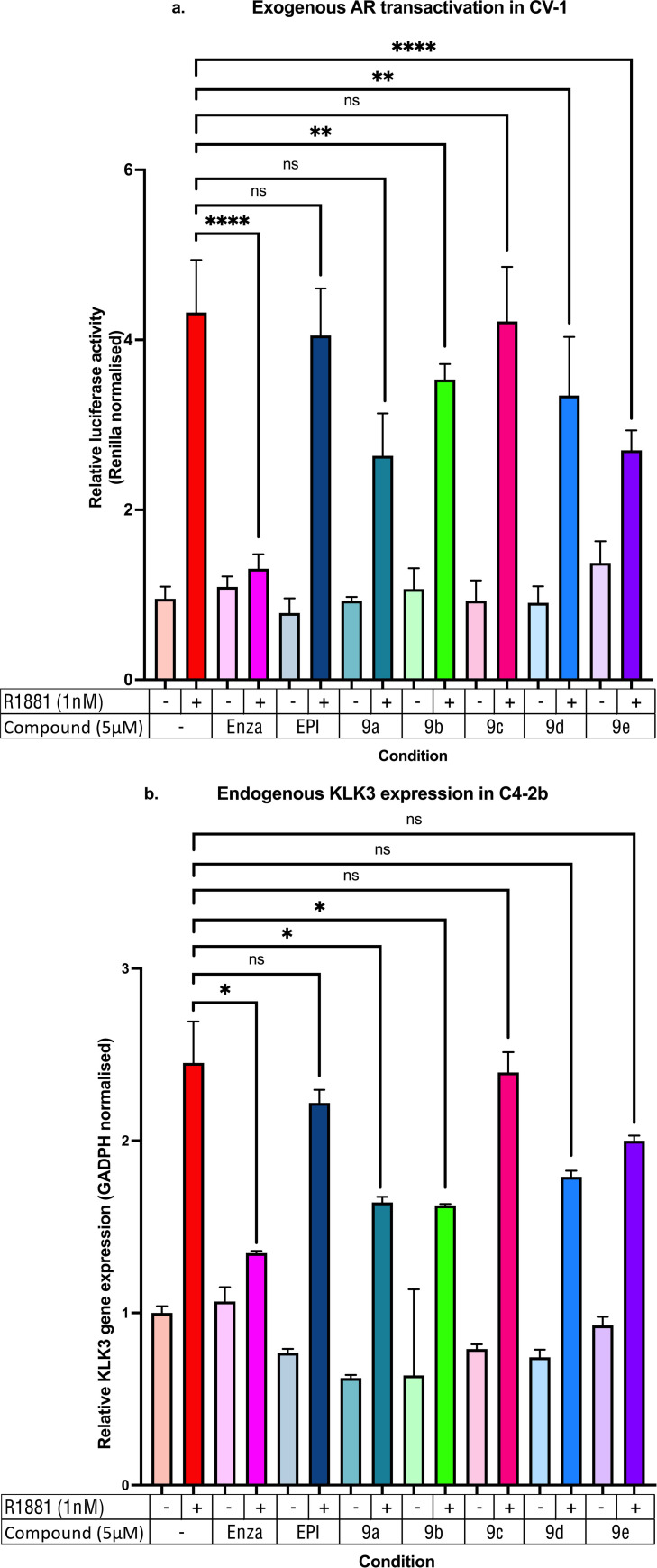
a) Exogenous AR transactivation in CV‐1: Dual luciferase reporter assays performed with AR‐FL‐Gal_4_ constructs. b) Endogenous KLK2 expression in C4‐2b: RT‐qPCR experiments performed for the quantification of the KLK3 transcript. Experiments performed in 3 biological replicates and 3 technical replicates. *p<0.05, **p<0.01, ***p<0.001, ns=non‐significant.

Results show that 9b, 9d and 9e significantly inhibit the transcriptional activity of a Gal4‐full length androgen receptor (Gal4‐AR‐FL) construct (Figure 4a), further confirming that these compounds can inhibit the AR transcriptional activity. Compound **9 c** was not able to inhibit gene transcription, suggesting its effects on cell growth are not AR mediated. Overall, the compounds were less potent than enzalutamide in engaging the luciferase reporter, which we hypothesise could be due to membrane permeability issues.

#### Effects on the transcriptional activity of endogenous AR

With these results in hand, we focused on investigating the effect of compounds **9 b**–**9 e** on endogenous AR mediated transcription. The KLK3 is a gene tightly regulated by the androgen receptor, with its transcript level highly sensitive to the AR activity^[33]^ Hence, RT‐qPCR experiments were performed in order to investigate the KLK3 gene expression levels at a concentration of 5 μM for the drugs, in C4‐2b cells. The synthetic androgen, R1881, was used at a concentration of 1 nM. Enzalutamide acted as a positive control at a concentration of 5 μM, while EPI‐001 was not able to inhibit the KLK3 gene transcription at the tested concentration, in accordance with previous findings.^[32]^ Compound **9 b** was able to significantly inhibit the KLK3 gene transcription at the tested concentrations (Figure 4b). While compound 9e failed to induce a statistically significant change in KLK3 gene expression (p=0.1186), compound **9 b** was able to cause a significant change (p=0.0397). Overall, the compounds were less potent than enzalutamide in activating downstream transcription, which we hypothesise could be due to membrane permeability issues.

## Discussion

Following the results of the robust biological assays, compound **9 b** has emerged as the lead candidate compound from our study. With an LC_50_ of 1.6 μM in C4‐2b cells and negligible toxicity in PC‐3 cells (>75 % cell viability at 10 μM), compound **9 b** was able to inhibit AR mediated gene transcription in the luciferase assay, proving its direct target engagement with the AR. Moreover, the compound was able to significantly inhibit the transcription of the KLK3 gene, further proving that its effects are specifically mediated via AR inhibition. We hypothesise that the marked improvement in cell toxicity could be due to an entropic effect, whereby the linker increases the effective local concentration of the second inhibitor that binds to the androgen receptor. This theory is supported especially by the fact that the dual inhibitors outperform an equimolar cocktail of EPI‐001 and enzalutamide by a factor of 80.

## Conclusions

We have synthesised a novel class of a previously unreported type of AR inhibitors. An 18 to 53 fold improvement in cell killing potency (LC_50_) was obtained for all tested compounds. Cellular toxicity for **9 b**–**9 e** was proven to be induced via AR inhibition given their lack of toxicity towards PC‐3 cells. Compound **9 e** outperformed all tested compounds in target engagement dual luciferase reporter assays (p<0.0001), indicating its potent and selective mechanism of action. KLK3 RT‐qPCR assays found compound **9 b** to be the most promising candidate (p=0.0397).

Altogether, these results suggest that the architecture of compound **9 b** is promising for further development and investigation. In summary, this research demonstrates the synthesis and characterisation of compound **9 b** as a first‐in‐class AR inhibitor that is more effective and specific at inhibiting the growth of C4‐2b cells compared to standard of care drug enzalutamide. This research has high potential to lead the development of next generation AR signalling inhibitors for otherwise incurable aggressive PCa.

## Experimental Section

### Chemical synthesis

#### General experimental techniques

All experiments were performed in oven‐dried glassware and under an atmosphere of nitrogen, unless stated otherwise. Commercial starting materials were used without further purification. Dry solvents were distilled from mixtures containing CaH_2_ or LiAlH_4_ as drying agents. Yields refer to spectroscopically and chromatographically pure compounds unless otherwise specified. Analytical thin layer chromatography (TLC) was carried out on glass Merck Kieselgel 60 F254 plates. The plates were visualised under direct UV irradiation (254 nm). R_*f*_ values are quoted to the nearest 0.1. Preparative thin layer chromatography was performed on commercially available Analtech plates. Flash column chromatography was undertaken on silica gel 60 (230‐.400 mesh) under a positive air pressure. The eluent systems are reported as % (v/v) of the solvent components.

Reverse phase column chromatography was carried out using a Combiflash Rf200 automated chromatography system with Redisep® reverse‐phase C18‐silica flash columns (20‐40 μm). Preparative high‐performance liquid chromatography (HPLC) was performed on an Agilent 1260 infinity machine. The samples were eluted using a Supelcosil ABZ+PLUS column (250 mm×21.2 mm, 5 μm). The used linear gradient (for 20 min and a flow rate of 20 mL/min) was: solvent A – 0.1 % (v/v) TFA in water, solvent B – 0.05 % (v/v) TFA in MeCN. The diodes used the wavelength of 220 nm and 254 nm in order to detect absorbance.

Infrared spectra were recorded on a Perkin Elmer Spectrum One FT‐IR spectrometer which is fitted with attenuated total reflectance (ATR) sampling accessory. The absorption maxima (ν) higher than 500 cm^−1^ are quoted in wavenumbers (cm^−1^) and are presented with the aid of abbreviations: w, weak; m, medium; s, strong; br, broad. Data are reported as: wavenumbers, assignment.

^1^H NMR spectra were recorded under an internal deuterium lock at rt on Bruker Advance III HD (400 MHz, 500 MHz, 700 MHz; Smart probe). Assignments are supported by ^1^H‐^1^H COSY, ^1^H‐^13^C HSQC and ^1^H‐^13^C HMBC spectra. Chemical shifts (**δ**) are given in ppm quoted to the nearest 0.01 ppm (**δ_H_**). The residual solvent peaks are 7.26 for CDCl_3_, 5.32 for CD_2_Cl_2_, 3.31 for CD_3_OD and 2.51 for (CD_3_)_2_SO. Coupling constants for mutually coupling protons are reported in Hertz, rounded to the nearest 0.1 Hz. Data are reported as: chemical shift, multiplicity (br, broad; s, singlet; d, doublet; t, triplet; q, quartet; m, multiplet; or a combination of them), coupling constants, number of nuclei. Spectra were processed using TopSpin v.4.0.6(Bruker). Carbon magnetic resonance spectra were recorded using an internal deuterium lock at rt on Bruker Avance III HD (101 MHz) with broadband proton decoupling. Chemical shifts (**δ_C_**) are quoted to the nearest 0.1 ppm and the solvent reference peaks (in ppm) are 77.2 (CDCl_3_), 53.5 (CD_2_Cl_2_), 49.1 (CD_3_OD), 33.0 (CD_3_)_2_SO). Fluorine magnetic resonance spectra were recorded using an internal deuterium lock at rt on Bruker Avance III HD (101 MHz) with broadband proton decoupling. Chemical shifts (**δ_C_**) are quoted to the nearest 0.1 ppm. For fluorine containing compounds, data are reported as: chemical shift, multiplicity, coupling constant. For the other compounds, data are reported as chemical shifts. Spectra were processed using TopSpin v.4.0.6(Bruker).

High resolution mass spectrometry (HRMS) measurements were performed on a Waters LCT Premier Time of Flight mass spectrometer, with errors within ±5 ppm.

#### General synthetic procedures

##### General synthetic procedure for 5 b–5 e

To a mixture of corresponding diol (1 eq., 3.1 M) in THF cooled to 0 °C was added NaH (0.4 eq.) in small portions. A solution of propargyl bromide in toluene (6.6 M, 0.62 eq.) was added dropwise at 0 °C. The mixture was subsequently stirred at room temperature for 24 h, and then H_2_O (30 mL) was added dropwise to the mixture at 0 °C. The resulting mixture was extracted with CH_2_Cl_2_, dried with Na_2_SO_4_ and the solvent subsequently removed *in vacuo*. The resulting alkynes were purified by silica gel chromatography (CH_2_Cl_2_ /EtOAc and acetone/hexane mixtures) to yield the corresponding alkynes.

##### General synthetic procedure for 3 b–3 e

To a solution of **2** in CH_3_CN (0.26 M) was added corresponding alkyne (6.6 eq.) and the mixture stirred for 10 minutes. Er(OTf)_3_ (0.2 eq.) was subsequently added and the mixture was stirred at room temperature for 24 h. The solvent subsequently removed under a stream on N_2_. The mixture was purified by reverse phase column chromatography (H_2_O/MeCN 9 : 1–1 : 9) to yield the corresponding alkynes.

##### General synthetic procedure for 9 a–9 e

Corresponding alkyne (1.1 equivalents) was dissolved in CH_2_Cl_2_/tBuOH/H_2_O 1 : 1 : 1 (11.5 mM) along with azide (1 eq.). In a separate flask, CuSO_4_×5 H_2_O (1 eq.) and THPTA (1.2 eq.) were dissolved in CH_2_Cl_2_/tBuOH/H_2_O 1 : 1 : 1 (11.5 mM). The reaction was subsequently stirred at room temperature for 24 h. The solvent was removed under a stream of nitrogen and the residue dissolved in CH_2_Cl_2_ (5 mL) and subsequently filtered. The residue was purified by reverse phase preparative HPLC (0.1 % TFA H_2_O/MeCN 2 : 3–1 : 9) to yield compounds **9 a**–**9 e**.

#### Synthetic procedures

##### 1‐chloro‐3‐(4‐(2‐(4‐(oxiran‐2‐ylmethoxy)phenyl)propan‐ 2‐yl)phenoxy)propan‐2‐ol (2)

To a solution of 1 (5.32 g; 15.6 mmol) in CH_3_CN (12 mL) was added CeCl_3_×7 H_2_O (2.92 g; 7.8 mmol) and the mixture refluxed for 3.5 h. The resulting white paste was concentrated *in vacuo*. The crude product was purified by flash column chromatography (DCM/EtOAc 99 : 1–95 : 5) to yield **2** (881 mg, 2.34 mmol, 15 %) as a transparent viscous liquid. R_*f*_=0.6 (8 : 2 DCM : EtOAc) **IR**: ν/cm^−1^=3489 (br, O−H), 2967 (w, C−H), 2928 (w, C−H), 2874 (w, C−H), 1606 (s), 1582 (s), 1507 (s), 1296 (w), 1232 (s), 1181 (s), 1034 (s), 827 (s), 748 (w).

^**1**^ **H NMR** (400 MHz, d_6_‐DMSO) **δ_H_** 7.10 (d, *J*=8.3 Hz, 4H), 6.84 (d, *J*=8.8 Hz, 2H), 6.83 (d, *J*=8.8 Hz, 2H), 5.55 (d, *J*=5.3 Hz, 1H), ), 4.26 (dd, *J*_1_=11.3 Hz, *J*_2_=2.5 Hz, 1H), 4.06–3.98 (m, 1H), 3.93 (d, *J*=5.2 Hz, 1H), 3.77 (dd, *J*_1_=11.4 Hz, *J*_2_=6.5 Hz, 1H), 3.73 (dd, *J*_1_=11.5 Hz, *J*_2_=4.6 Hz, 1H), 3.65 (dd, *J*_1_=11.2 Hz, *J*_2_=5.5 Hz, 1H), 3.31–3.27 (m, 1H), 2.83 (dd, *J*_1_=*J*_2_=4.65 Hz, 1H), 2.77 (dd, *J*_1_=5.0 Hz, *J*_2_=2.6 Hz, 1H), 1.57 (s, 6H). ^**13**^ **C‐NMR** (126 MHz, d_6_‐DMSO) **δ_C_** 156.6, 156.5,143.4, 143.3, 127.6, 114.1, 69.1, 69.0, 68.8, 50.0, 46.9, 44.0, 41.6, 31.1. Data are in accordance with literature.^[34]^

##### 1‐chloro‐3‐(4‐(2‐(4‐(2‐hydroxy‐3‐(prop‐2‐yn‐1‐yloxy)propoxy)phenyl)propan‐2‐yl)phenoxy)propan‐2‐ol (3 a)

**2** (100 mg; 0.262 mmol, 1 eq.) was added to propargyl alcohol (4 mL) and the mixture stirred for 10 minutes. Er(OTf)_3_ (36 mg; 0.05 mmol; 0.2 eq.) was subsequently added and the mixture was stirred at room temperature for 24 h. The solvent subsequently removed under a stream on N_2_. The mixture was purified by reverse phase column chromatography (H_2_O/MeCN 9 : 1–1 : 9) to yield **3 a** (51 mg; 0.12 mmol; 45 %) as a colourless oil. **IR**: ν/cm^−1^=3413 (br, O−H), 3288 (m), 2965 (m, C−H), 2928 (m, C−H), 2870 (s), 1607 (m), 1581 (w), 1508 (s), 1459 (m), 1295 (m), 1243 (s), 1181 (s), 1085 (s), 1037(s), 941 (w), 911 (w), 828 (s), 642 (w), 637 (w). ^**1**^ **H NMR** (400 MHz, CDCl_3_) **δ_H_** 7.13 (d, *J=*8.9 Hz, 4H), 7.11 (d, *J=*8.9 Hz, 4H), 6.79(d, *J=*8.6 Hz, 2H), 4.20 (d, *J=*2.4 Hz, 2H), 4.17 (quintet, *J=*5.3 Hz, 1H), 4.15–4.11 (m, 1H), 4.04 (m, 2H), 3.99 (d, *J=*5.0 Hz, 1H), 3.98 (d, *J=*6 Hz, 1H), 3.75 (dd, *J*_1_ *=*11.6 Hz, *J*_2_ *=*5.4 Hz, 1H), 3.72 (dd, *J*_1_ *=*11.6 Hz, *J*_2_ *=*5.4 Hz, 1H), 3.69 (dd, *J*_1_ *=*11.6 Hz, *J*_2_ *=*5.4 Hz, 1H), 3.66 (dd, *J*_1_ *=*11.6 Hz, *J*_2_ *=*5.4 Hz, 1H), 2.43 (t, *J=*2.4 Hz, 1H), 1.61 (s, 6H). ^**13**^ **C‐NMR** (400 MHz, CDCl_3_ ) **δ_C_** 156.3, 156.0, 143.9, 143.5, 127.8, 127.7, 113.9×2, 79.3, 74.9, 70.8, 69.9, 69.0, 68.8, 68.4, 58.7, 46.0, 41.7, 31.0. **HRMS** (ESI+): m/*z* [M+H]^+^ calculated for C_24_H_30_ClO_5_: **433.1782**; found **433.1798**. error: 3.7 ppm.

##### 1‐chloro‐3‐(4‐(2‐(4‐(2‐hydroxy‐3‐(2‐(prop‐2‐yn‐1‐yloxy)ethoxy)propoxy)phenyl)propan‐2‐yl)phenoxy)propan‐2‐ol (3 b)

Colourless oil (62 mg; 0.13 mmol; 33 %). **IR**: ν/cm^−1^=3418 (br, O−H), 3286 (m), 2968 (m), 2926 (m), 2871 (s), 1607 (m), 1581 (w), 1508 (s), 1459 (m), 1383 (w), 1361 (w), 1294 (w), 1245 (s), 1182 (s), 1087 (s), 1035(s), 1011 (m), 946 (w), 828 (s), 737 (w). ^**1**^ **H NMR** (400 MHz, CDCl_3_) **δ_H_** 7.15 (d, *J=*8.8 Hz, 2H), 7.13 (d, *J=*8.7 Hz, 2H), 6.81 (d, *J=*8.6 Hz, 4H), 4.19 (quintet, *J=*5.3 Hz, 1H), 4.12 (d, *J=*2.4 Hz, 2H), 4.15–4.11 (m, 1H), 4.06 (dd, *J*_1_ *=*5.3 Hz, *J*_2_ *=*4.0 Hz, 2H), 4.00 (d, *J=*5.0 Hz, 1H), 3.99 (d, *J=*6 Hz, 1H), 3.77 (dd, *J*_1_ *=*11.6 Hz, *J*_2_ *=*5.4 Hz, 1H), 3.73–3.68 (m, 5H), 3.63 (dd, *J*_1_ *=*11.6 Hz, *J*_2_ *=*5.7 Hz, 1H), 2.44 (t, *J=*2.4 Hz, 1H), 2.60 (brs, 2H), 1.63 (s, 6H).^**13**^ **C‐NMR** (126 MHz, CDCl_3_) **δ_C_** 156.4, 156.0, 143.9, 143.4, 127.8, 127.7, 113.9×2, 79.4, 74.7, 72.3, 70.6, 69.9, 69.0×2, 68.8, 68.4, 58.4, 46.0, 41.7, 31.0.

**HRMS** (ESI+): m/*z* [M+Na]^+^ calculated for C_26_H_33_ClO_6_Na: **499.1863**; found **499.1853**. error: −2.0 ppm.

##### 1‐chloro‐3‐(4‐(2‐(4‐(2‐hydroxy‐3‐(2‐(2‐(prop‐2‐yn‐1‐yloxy)ethoxy)ethoxy)propoxy)phenyl)propan‐2‐yl)phenoxy)propan‐2‐ol (3 c)

Colourless oil (116 mg; 0.22 mmol; 60 %) **IR**: ν/cm^−1^=3421 (br, O−H), 3287 (m), 2869 (s), 1607 (m), 1581 (w), 1508 (s), 1459 (m), 1383 (w), 1361 (w), 1295 (w), 1245 (s), 1182 (s), 1086 (s), 1037(s), 1035 (s), 941 (w), 829 (s), 737 (w), 671 (w). ^**1**^ **H NMR** (400 MHz, CDCl_3_) **δ_H_** 7.11 (d, *J=*8.8 Hz, 2H), 7.11 (d, *J=*8.7 Hz, 2H), 6.79 (d, *J=*8.6 Hz, 4H), 4.19 (quintet, *J=*5.3 Hz, 1H), 4.12 (d, *J=*2.4 Hz, 2H), 4.15–4.11 (m, 1H), 4.04 (t, *J=*4.5 Hz, 2H), 3.98 (d, *J=*5.0 Hz, 2H), 3.76 (dd, *J*_1_ *=*11.6 Hz, *J*_2_ *=*5.4 Hz, 1H), 3.72–3.58 (m, 11H), 2.39 (t, *J=*2.4 Hz, 1H), 2.39 (brs, 2H), 1.61 (s, 6H). ^**13**^ **C‐NMR** (126 MHz, CDCl_3_) **δ_C_** 156.4, 156.0, 143.9, 143.4, 127.8, 127.7, 113.9×2, 79.6, 74.6, 72.3, 70.8, 70.6, 70.4, 70.3, 69.9, 69.0, 68.8, 68.4, 58.4, 46.0, 41.7, 31.0.

**HRMS** (ESI+): m/*z* [M+H]^+^ calculated for C_28_H_38_ClO_7_: **504.2306**; found **521.2307**. error: 0.2.

##### 1‐(4‐(2‐(4‐(3‐chloro‐2‐hydroxypropoxy)phenyl)propan‐2‐ yl)phenoxy)‐4,7,10,13,16‐pentaoxanonadec‐18‐yn‐2‐ol (3 d)

Colourless oil (68 mg; 0.11 mmol; 29 %). IR: ν/cm^−1^=3415 (br), 3270 (m,), 2868 (s), 1607 (m), 1581 (w), 1508 (s), 1459 (m), 1349 (w), 1294 (w), 1246 (s), 1182 (s), 1091 (s), 1037(s), 945 (w), 829 (s), 737 (w). ^1^H NMR (700 MHz, CDCl_3_) δ_H_ 7.11 (d, *J=*8.9 Hz, 2H), 7.11 (d, *J=*8.9 Hz, 2H), 6.79 (d, *J=*8.6 Hz, 4H), 4.17 (quintet, *J=*5.3 Hz, 1H), 4.16 (d, *J=*2.4 Hz, 2H), 4.15–4.11 (m, 1H), 4.04 (m, 2H), 3.98 (d, *J=*5.0 Hz, 2H), 3.75 (dd, *J*_1_ *=*11.6 Hz, *J*_2_ *=*5.4 Hz, 1H), 3.72–3.58 (m, 19H), 2.52 (brs, 2H), 2.40 (t, *J=*2.4 Hz, 1H), 1.61 (s, 6H).^13^C‐NMR (700 MHz, CDCl_3_) δ_C_ 156.4, 156.0, 143.9, 143.3, 127.8, 127.7, 113.9×2, 79.7, 74.5, 72.4, 70.8, 70.58, 70.56, 70.54, 70.53, 70.52, 70.51, 70.4, 69.9, 69.0×2, 68.8, 68.4, 58.4, 46.0, 41.7, 31.0.

**HRMS** (ESI+): m/*z* [M+H]^+^ calculated for C_32_H_46_ClO_9_: **608.2752**; found **608.2762**. error: 1.6 ppm.

##### 1‐chloro‐3‐(4‐(2‐(4‐(2‐hydroxy‐3‐(4‐(prop‐2‐yn‐1‐yloxy)butoxy)propoxy)phenyl)propan‐2‐yl)phenoxy)propan‐2‐ol (3 e)

Colourless oil (38 mg; 75 μmol; 29 %)

**R**_***f***_=0.2 (8 : 2 DCM:EtOAc) ^**1**^ **H NMR** (400 MHz, CDCl_3_) **δ_H_** 7.14 (d, *J=*8.8 Hz, 2H), 7.13 (d, *J=*8.7 Hz, 2H), 6.81 (d, *J=*8.6 Hz, 4H), 4.19 (quintet, *J=*5.3 Hz, 1H), 4.12 (d, *J=*2.4 Hz, 2H), 4.15–4.11 (m, 1H), 4.06 (dd, *J*_1_ *=*5.3 Hz, *J*_2_ *=*4.0 Hz, 2H), 4.00 (d, *J=*5.0 Hz, 1H), 3.99 (d, *J=*6 Hz, 1H), 3.77 (dd, *J*_1_ *=*11.6 Hz, *J*_2_ *=*5.4 Hz, 1H), 3.71 (dd, *J*_1_ *=*11.6 Hz, *J*_2_ *=*5.7 Hz, 1H), 3.61 (dd, *J*_1_ *=*10.1 Hz, *J*_2_ *=*4.6 Hz, 1H), 3.58–3.50 (m, 5H), 2.71 (t, *J=*2.4 Hz, 1H), 2.34 (brs, 2H), 1.70–1.65 (m, 4H), 1.63 (s, 6H). ^**13**^ **C‐NMR** (126 MHz, CDCl_3_) **δ_C_** 156.4, 156.0, 143.9, 143.4, 127.9, 127.7, 113.9×2, 79.9, 74.2, 71.5, 71.2, 69.9, 69.8, 69.1, 68.9, 68.4, 58.0, 46.0, 41.7, 31.0, 26.3, 26.2.

**HRMS** (ESI+): m/*z* [M+H]^+^ calculated for C_28_H_38_ClO_6_: **504.2279**; found **504.2291**. error: 2.4 ppm.

##### 2‐(prop‐2‐yn‐1‐yloxy)ethan‐1‐ol (5 b)

Purified by silica gel chromatography (CH_2_Cl_2_/EtOAc 9 : 1–3 : 7) to yield **5 b** (318 mg; 3.68 mmol; 32 %) as a yellow liquid. **R**_***f***_=0.2 (8 : 2 DCM:EtOAc) **IR**: ν/cm^−1^=3401 (br, O−H), 3294 (m), 2874 (s), 1607 (m), 1581 (w), 1508 (s), 1459 (m), 1295 (w), 1245 (s), 1182 (s), 1087 (s), 1035(s), 949 (w), 828 (s), 736 (w), 678 (w).

^**1**^ **H NMR** (700 MHz, CDCl_3_) **δ_H_** 4.18 (d, *J=*2.4 Hz, 2H), 3.75 (d, *J=*5.8 Hz, 1H), 3.75 (d, *J=*4.8 Hz, 1H), 3.63 (d, *J=*3.6 Hz, 1H), 3.62 (d, *J=*5.5 Hz, 1H), 2.39 (t, *J=*2.4 Hz, 1H), 2.01 (brs, 1H).

^**13**^ **C‐NMR** (700 MHz, CDCl_3_) **δ_C_** 79.4, 74.7, 71.2, 61.7, 58.4.

Data are in accordance with literature.^[35]^

##### 2‐(2‐(prop‐2‐yn‐1‐yloxy)ethoxy)ethan‐1‐ol (5 c)

Purified by silica gel chromatography (CH_2_Cl_2_/EtOAc 9 : 1–3 : 7) to yield **5 c** as a yellow liquid (693 mg; 4.81 mmol; 41 %). **R**_***f***_=0.2 (8 : 2 DCM:EtOAc). **IR**: ν/cm^−1^=3393 (br, O−H), 3287 (m), 2920 (s), 1455 (m), 1352 (m), 1234 (m), 1063 (s), 921 (m), 887 (m), 840 (m), 755 (m). ^**1**^ **H NMR** (700 MHz, CDCl_3_) **δ_H_** 4.17 (d, *J=*2.4 Hz, 2H), 3.75–3.65 (m, 6H), 3.61–3.56 (m, 2H), 2.42 (t, *J=*2.4 Hz, 1H), 2.03 (brs, 1H). ^**13**^ **C‐NMR** (700 MHz, CDCl_3_) **δ_C_** 79.4, 74.7, 72.5, 70.2, 69.1, 61.7, 58.4. Data are in accordance with literature.^[36]^

##### 3,6,9,12‐tetraoxapentadec‐14‐yn‐1‐ol (5 d)

Purified by reverse phase chromatography (H_2_O/MeCN 9 : 1–1 : 9) to yield **5 d** as a yellow liquid (509 mg; 2.19 mmol; 19 %).

**R**_***f***_=0.2 (8 : 2 DCM:EtOAc)

**IR**: ν/cm^−1^=3465 (br, O−H), 3246 (m, ‐C≡C**H**), 2867 (s, ‐**C≡C**H), 1455 (m), 1349 (m), 1288 (w), 1247 (w), 1092 (s), 1032 (m), 919 (w), 884 (w), 840 (w).

^**1**^ **H NMR** (700 MHz, CDCl_3_) **δ_H_** 4.17 (d, *J=*2.4 Hz, 2H, Hd’), 3.75–3.56 (m, 16H, ‐**C**H_2_‐O), 2.61 (brs, 1H, OH), 2.40 (t, *J=*2.4 Hz, 1H, Ha’).

^**13**^ **C‐NMR** (700 MHz, CDCl_3_ ) **δ_C_** 79.6 (‐**C≡**CH), 74.5 (Ca’), 72.5, 70.6 (×3), 70.5, 70.4, 70.3, 69.1 (8×−**C**H_2_−O), 58.4 (Cd’).

Data are in accordance with literature.^[37]^

##### 2‐(2‐(prop‐2‐yn‐1‐yloxy)ethoxy)ethan‐1‐ol (5 e)

Purified by silica gel chromatography (CH_2_Cl_2_/EtOAc 9 : 1–4 : 6) to yield **5 e** as a yellow liquid (251 mg; 1.95 mmol; 17 %). **R**_***f***_=0.2 (8 : 2 DCM:EtOAc). ^**1**^ **H NMR** (400 MHz, CDCl_3_) **δ_H_** 4.14 (s, 2H), 3.65 (t, *J=*5.5 Hz, 2H), 3.56 (t, *J=*5.5 Hz, 2H), 2.42 (s, 1H), 1.90 (brs, 1H), 1.72–1.64 (m, 4H) ^**13**^ **C‐NMR** (400 MHz, CDCl_3_ ) **δ_C_** 79.7, 74.3, 70.0, 62.6, 58.1, 29.8, 26.2.

Data are in accordance with literature.^[38]^

##### 4‐(3‐(4‐cyano‐3‐(trifluoromethyl)phenyl)‐5,5‐dimethyl‐4‐oxo‐2‐thioxoimidazolidin‐1‐yl)‐2‐fluorobenzoic acid (7)

Enzalutamide (60 mg; 0.129 mmol) was dissolved in concentrated HCl 36.5 % (1.5 mL) and the mixture heated in a sealed tube at 120 °C for 72 h. The resulting mixture was extracted with EtOAc (3×5 mL), the resulting mixture concentrated *in vacuo* and purified by silica gel chromatography (CH_2_Cl_2_/MeOH 99 : 1–95 : 5 and then CH_2_Cl_2_/MeOH 95 : 5+1 % AcOH) to yield **7** (24 mg; 0.052 mmol; 40 %) as a white powder.

**R**_***f***_=0.2 (95 : 5 DCM:MeOH). ^**1**^ **H NMR** (400 MHz, CDCl_3_) **δ_H_** 8.22 (dd, *J_1_* =*J_2_* =7.8 Hz, 1H), 8.03 (d, *J=*8.4 Hz, 1H), 7.97 (s, 1H), 7.85 (d, *J=*8.4 Hz, 1H), 7.26 (d, *J=*7.8 Hz, 1H), 7.22 (d, *J=*10.8 Hz, 1H), 1.65 (s, 6H). ^**13**^ **C‐NMR** (126 MHz, CDCl_3_ ) **δ_C_** 179.7, 174.3, 165.7, 162.6 (d, *J=*265 Hz), 141.3, 136.7, 135.3, 133.9, 133.8 (q, *J=*34 Hz), 132.1, 127.1(q, *J*=5 Hz), 125.7, 120.5, 119.0 (d, *J=*24 Hz), 114.7, 110.5 (q, *J=*2 Hz), 66.7, 23.9. ^**19**^ **F‐NMR** (376 MHz, CDCl_3_) −62.0, −104.2. Data are in accordance with literature.^[16]^

##### *N*‐(2‐(2‐(2‐(2‐azidoethoxy)ethoxy)ethoxy)ethyl)‐4‐(3‐(4‐cyano‐ 3‐(trifluoromethyl)phenyl)‐5,5‐dimethyl‐4‐oxo‐2‐thioxoimidazolidin‐1‐yl)‐2‐fluorobenzamide (8)

**7** (59 mg ; 0.13 mmol; 1 eq.) was dissolved in DMF (527 μL) and DCC (43 mg; 0.21 mmol, 1.7 eq.) and HOAt (0.6 M in DMF, 352 μL, 0.21 mmol, 1.7 eq.) were added. The mixture was stirred at room temperature for 30 minutes before a further portion of DCC (28 mg; 0.14 mmol; 1.1 eq.) was added. After 30 min, 2‐(2‐(2‐(2‐azidoethoxy)ethoxy)ethoxy)ethan‐1‐amine (29 mg, 26 μL, 0.13 mmol, 1 eq.) in DMF (0.85 mL) was added, and the reaction stirred at room temperature for 24 h. The solvent was removed under a stream of nitrogen and the residue dissolved in CH_2_Cl_2_ (5 mL) and subsequently filtered. The solvent was removed *in vacuo* and subsequently purified by silica gel chromatography (CH_2_Cl_2_/EtOAc 9 : 1–1 : 1) to yield **8** (38 mg; 0.06 mmol; 45 %) as a transparent foam. **R**_***f***_=0.2 (8 : 2 DCM : EtOAc). **IR**: ν/cm^−1^=3489 (br, O−H), 2967 (w, C−H), 2928 (w, C−H), 2874 (w, C−H), 1606 (s), 1582 (s), 1507 (s). ^**1**^ **H NMR** (400 MHz, CDCl_3_) **δ_H_** 8.23 (dd, *J_1_* =*J_2_* =8.3 Hz, 1H), 8.01 (d, *J=*8.2 Hz, 1H), 7.97 (s, 1H), 7.84 (d, *J=*8.3 Hz, 1H), 7.25 (d, *J=*8.3 Hz, 1H), 7.22 (d, *J=*10.6 Hz, 1H), 7.17 (brs, NH), 3.74‐3.67 (m, 14H), 3.38 (t, *J=*9.9 Hz, 2H) 1.63 (s, 6H). ^**13**^ **C‐NMR** (126 MHz, CDCl_3_ ) **δ_C_** 179.7, 174.4, 162.9, 160.3 (d, *J=*251 Hz) 141.3 (d, *J=*11.0 Hz), 136.8, 135.3, 133.7 (q, *J=*34 Hz), 133.2 (d, *J=*6.2 Hz), 132.2, 127.1 (q, *J*=4.8 Hz), 126.0 (d, *J*=3.3 Hz), 123.0 (d, *J*=12.2 Hz), 121.8 (q, *J=*274 Hz), 117.9 (d, *J=*26 Hz), 114.7, 110.5 (q, *J=*2.1 Hz), 70.7, 70.6, 70.6, 70.4, 70.0, 69.5, 66.6, 50.6, 39.9, 23.9. ^**19**^ **F‐NMR** (376 MHz, CDCl_3_) −62.0, −110.3.

**9 a**. Transparent foam (31 mg; 29 μmol; 62 %)

**IR**: ν/cm^−1^=3459 (br), 2922 (m, C−H), 2236 (w), 2103 (w), 1758 (s), 1727 (s), 1656 (s), 1619 (s), 1579 (w), 1506 (s), 1439 (s), 1412 (s), 1310 (s), 1247 (s), 1219 (s), 1180 (s), 1135 (s), 1037 (s), 909 (s), 830 (s), 813 (m), 729 (s), 678 (w).

^**1**^ **H NMR** (400 MHz, CD_2_Cl_2_) **δ_H_** 8.12 (dd, *J_1_* =*J_2_* =8.4 Hz, 1H), 8.01 (d, *J=*8.3 Hz, 1H), 7.98 (d, *J=*1.9 Hz, 1H), 7.86 (dd, *J*_1_ *=*8.2 Hz, *J*_2_ *=*1.9 Hz, 1H), 7.83 (s, 1H), 7.25 (dd, *J*_1_ *=*8.3 Hz, *J*_2_ *=*1.7 Hz, 1H), 7.22 (brs, 1H), 7.18 (dd, *J*_1_ *=*10.6 Hz, *J*_2_ *=*1.8 Hz, 1H), 7.13 (d, *J=*8.7 Hz, 2H), 7.12 (d, *J=*8.7 Hz, 2H), 6.81 (d, *J=*8.7 Hz, 2H), 6.79 (d, *J=*8.7 Hz, 2H), 4.68 (s, 2H), 4.53 (t, *J=*4.9 Hz, 2H), 4.17 (quintet, *J=*5.3 Hz, 1H), 4.15–4.10 (m, 1H), 4.06 (brs, 2H), 4.04 (d, *J=*5.4 Hz, 2H), 3.95 (t, *J=*5.0 Hz, 2H), 3.84 (t, *J=*5.0 Hz, 2H), 3.77 (dd, *J*_1_ *=*11.7 Hz, *J*_2_ *=*5.3 Hz, 1H), 3.75–3.60 (m, 15H), 1.62 (s, 6H), 1.58 (s, 6H).

^**13**^ **C‐NMR** (126 MHz, CD_2_Cl_2_) **δ_C_** 180.0, 174.6, 162.8 (d, *J=*3.0 Hz), 160.4 (d, *J=*251 Hz), 156.5, 156.3, 143.9, 143.6, 139.3 (d, *J=*10.8 Hz), 137.2, 135.5 (C_f_), 133.2 (q, *J=*33.6 Hz), 132.8 (d, *J=*3.3 Hz), 132.5 (d, *J=*0.8 Hz), 127.8, 127.7, 127.2 (q, *J*=4.95 Hz), 126.4 (d, *J*=3.4 Hz), 124.2, 122.9 (d, *J*=12.6 Hz), 122.0 (q, *J=*274 Hz), 118.1 (d, *J=*26 Hz), 114.9, 113.9×2, 110.2 (q, *J=*2.0 Hz), 71.8, 70.4, 70.3, 70.2×3, 69.9, 69.7, 69.2×2, 68.9, 66.8, 64.1, 50.8, 46.2, 41.7, 40.0, 30.7, 23.6.

^**19**^ **F‐NMR** (376 MHz, CDCl_3_) −62.9, −111.4. **HRMS** (ESI+): m/*z* [M+H]^+^ calculated for C_52_H_59_ClF_4_N_7_O_10_S: **1084.3669**; found **1084.3687**. error: 1.7 ppm. **HPLC purity** 98.2 %.

**9 b**.Transparent foam (35 mg; 31 μmol; 68 %). **R**_***f***_=0.2 (8 : 2 DCM:EtOAc). **IR**: ν/cm^−1^=3393 (br, O−H), 2927 (m, C−H), 2872 (m, C−H), 2210 (w), 1757 (s), 1727 (s), 1655 (s), 1619 (s), 1579 (w), 1506 (s), 1439 (s), 1412 (s), 1364 (w), 1310 (s), 1247 (s), 1219 (s), 1180 (s), 1132 (s), 1038 (s), 909 (s), 829 (s), 813 (s), 771 (w), 728 (s), 677 (w). ^**1**^ **H NMR** (400 MHz, CD_2_Cl_2_) **δ_H_** 8.13 (dd, *J_1_* =*J_2_* =8.4 Hz, 1H), 8.02 (d, *J=*8.3 Hz, 1H), 7.98 (d, *J=*1.9 Hz, 1H), 7.86 (dd, *J*_1_ *=*8.2 Hz, *J*_2_ *=*1.9 Hz, 1H), 7.83 (s, 1H), 7.25 (dd, *J*_1_ *=*8.3 Hz, *J*_2_ *=*1.7 Hz, 1H), 7.22 (brs, 1H), 7.18 (dd, *J*_1_ *=*10.6 Hz, *J*_2_ *=*1.8 Hz, 1H), 7.13 (d, *J=*8.7 Hz, 2H), 7.12 (d, *J=*8.7 Hz, 2H), 6.81 (d, *J=*8.7 Hz, 2H), 6.79 (d, *J=*8.7 Hz, 2H), 4.67 (s, 2H), 4.52 (t, *J=*4.9 Hz, 2H), 4.17 (quintet, *J=*5.3 Hz, 1H), 4.15–4.10 (m, 1H), 4.04 (d, *J=*5.4 Hz, 2H), 3.95 (t, *J=*5.0 Hz, 2H), 3.84 (t, *J=*5.0 Hz, 2H), 3.77 (dd, *J*_1_ *=*11.7 Hz, *J*_2_ *=*5.3 Hz, 1H), 3.75–3.60 (m, 19H), 2.17 (brs, 2H), 1.64 (s, 6H), 1.63 (s, 6H). ^**13**^ **C‐NMR** (126 MHz, CD_2_Cl_2_) **δ_C_** 180.0, 174.6, 162.8 (d, *J=*3.0 Hz), 160.4 (d, *J=*251 Hz), 156.5, 156.3, 143.9, 143.6, 139.3 (d, *J=*10.8 Hz), 137.2, 135.5, 133.2 (q, *J=*33.6 Hz), 132.8 (d, *J=*3.3 Hz), 132.5 (d, *J=*0.8 Hz), 127.8, 127.7, 127.2 (q, *J*=4.95 Hz), 126.4 (d, *J*=3.4 Hz), 124.2, 123.0 (d, *J*=12.6 Hz), 122.0 (q, *J=*274 Hz), 118.1 (d, *J=*26 Hz), 114.9, 113.90×2, 110.2 (q, *J=*2.0 Hz), 72.3, 70.7, 70.4, 70.3×3, 70.0, 69.8, 69.6, 69.2×2, 69.0, 68.7, 66.8, 63.8, 50.7, 46.2, 41.6, 40.0, 30.7, 23.6. ^**19**^ **F‐NMR** (376 MHz, CDCl_3_) −62.0, −110.4.

**HRMS** (ESI+): m/*z* [M+H]^+^ calculated for C_54_H_63_ClF_4_N_7_O_11_S: **1128.3931**; found **1128.3927**. error: −0.3 ppm. **HPLC purity** 100 %.

**9 c**. Transparent foam (7 mg; 5.5 μmol; 29 %) **IR**: ν/cm^−1^=3397 (br, O−H), 2923 (m), 2871 (m), 2322 (w), 2101 (w), 1757 (s), 1727 (s), 1659 (s), 1620 (s), 1538 (w), 1506 (s), 1439 (m), 1412 (s), 1311 (s), 1248 (m), 1180 (s), 1134 (s), 1034 (s), 1040 (m), 923 (w), 830 (w), 813 (m), 734 (s), 702 (w). ^**1**^ **H NMR** (400 MHz, CDCl_3_) **δ_H_** 8.18 (dd, *J_1_* =*J_2_* =8.3 Hz, 1H), 7.96 (d, *J=*8.2 Hz, 1H), 7.93 (d, *J=*1.5 Hz, 1H), 7.87 (s, 1H), 7.82 (dd, *J*_1_ *=*8.2 Hz, *J*_2_ *=*1.7 Hz, 1H), 7.21 (dd, *J*_1_ *=*8.3 Hz, *J*_2_ *=*1.7 Hz, 1H), 7.16 (dd, *J*_1_ *=*10.6 Hz, *J*_2_ *=*1.7 Hz, 1H), 7.16 (brs, 1H), 7.11 (d, *J=*8.7 Hz, 2H), 7.09 (d, *J=*8.7 Hz, 2H), 6.79 (d, *J=*8.7 Hz, 2H), 6.77 (d, *J=*8.7 Hz, 2H), 4.68 (s, 2H), 4.47 (t, *J=*4.9 Hz, 2H), 4.17 (quintet, *J=*5.3 Hz, 1H), 4.15–4.10 (m, 1H), 4.04 (t, *J=*5.0 Hz, 2H), 3.97 (d, *J=*5.5 Hz, 2H), 3.82 (t, *J=*5.0 Hz, 2H), 3.74 (dd, *J*_1_ *=*11.7 Hz, *J*_2_ *=*5.3 Hz, 1H), 3.77–3.45 (m, 23H), 2.9 (brs, 2H), 1.60 (s, 6H), 1.59 (s, 6H). ^**13**^ **C‐NMR** (126 MHz, CDCl_3_ ) **δ_C_** 179.8, 174.5, 162.2 (d, *J=*3.0 Hz), 160.3 (d, *J=*251 Hz), 156.4,156.0, 143.9, 143.4, 139.0 (d, *J=*10.7 Hz), 136.8, 135.3, 133.6 (q, *J=*33.6 Hz), 133.2 (d, *J=*3.3 Hz), 132.2, 127.8, 127.7, 127.1 (q, *J*=4.76 Hz), 126.1 (d, *J*=3.2 Hz), 123.0, 122.9 (d, *J*=12.6 Hz), 121.8 (q, *J=*274 Hz), 118.0 (d, *J=*26 Hz), 114.7, 113.92, 113.91, 110.4 (q, *J=*2.0 Hz), 72.5, 70.8, 70.54, 70.52, 70.50×10, 70.48, 69.9, 69.5, 69.1, 68.9, 68.4, 66.6, 63.8, 51.0, 46.0, 41.7, 39.9, 31.0, 23.9. ^**19**^ **F‐NMR** (376 MHz, CDCl_3_) −62.9, −110.4. **HRMS** (ESI+): m/*z* [M+H]^+^ calculated for C_56_H_67_ClF_4_N_7_O_12_S: **1172.3425**; found **1172.3435**. error: 0.9 ppm. **HPLC purity** 100 %.

**9 d**. Transparent foam (18 mg; 14.3 μmol; 63 %). ^**1**^ **H NMR** (400 MHz, CD_2_Cl_2_) **δ_H_** 8.15 (dd, *J_1_* =*J_2_* =8.4 Hz, 1H), 8.02 (d, *J=*8.3 Hz, 1H), 7.98 (d, *J=*1.9 Hz, 1H), 7.86 (dd, *J*_1_ *=*8.2 Hz, *J*_2_ *=*1.9 Hz, 1H), 7.75 (s, 1H), 7.25 (dd, *J*_1_ *=*8.3 Hz, *J*_2_ *=*1.7 Hz, 1H), 7.18 (dd, *J*_1_ *=*10.6 Hz, *J*_2_ *=*1.8 Hz, 1H), 7.13 (d, *J=*8.7 Hz, 2H), 7.12 (d, *J=*8.7 Hz, 2H), 7.12 (brs, 1H, NH), 6.81 (d, *J=*8.7 Hz, 2H), 6.79 (d, *J=*8.7 Hz, 2H), 4.60 (s, 2H), 4.48 (t, *J=*4.9 Hz, 2H), 4.17 (quintet, *J=*5.3 Hz, 1H), 4.14–4.08 (m, 1H), 4.03 (d, *J=*5.4 Hz, 2H), 3.96 (d, *J=*5.0 Hz, 2H), 3.83 (t, *J=*5.0 Hz, 2H), 3.77 (dd, *J*_1_ *=*11.7 Hz, *J*_2_ *=*5.3 Hz, 1H), 3.75–3.50 (m, 31H), 1.95 (brs, 2H), 1.62 (s, 6H), 1.59 (s, 6H). ^**13**^ **C‐NMR** (126 MHz, CD_2_Cl_2_) **δ_C_** 180.0, 174.6, 162.2 (d, *J=*3.0 Hz), 160.4 (d, *J=*251 Hz), 156.6, 156.3, 143.9, 143.5, 139.1 (d, *J=*10.8 Hz), 137.2, 135.5, 133.2 (q, *J=*33.6 Hz), 132.8 (d, *J=*3.3 Hz), 132.5 (d, *J=*0.8 Hz), 127.8, 127.7, 127.2 (q, *J*=4.95 Hz), 126.3 (d, *J*=3.4 Hz), 123.3, 123.2 (d, *J*=12.6 Hz), 122.0 (q, *J=*274 Hz), 118.1 (d, *J=*26 Hz), 114.9, 113.90×2, 110.2 (q, *J=*2.0 Hz), 72.3, 70.4, 70.30–70.10×15, 69.8, 69.4, 69.3, 68.78, 68.76, 68.7, 66.8, 64.2, 50.3, 46.3, 41.7, 40.0, 30.7, 23.6. ^**19**^ **F‐NMR** (376 MHz, CDCl_3_) −62.0 (CF_3_), −110.4 (ArC−F). **HRMS** (ESI+): m/*z* [M+H]^+^ calculated for C_60_H_75_ClF_4_N_7_O_14_S: **1260.4717**; found **1260.4738**. error: 1.7 ppm. **HPLC purity** 98.4 %.

**9 e**. Transparent foam (28 mg; 24.0 μmol; 42 %) **R**_***f***_=0.2 (8 : 2 DCM:EtOAc). **IR**: ν/cm^−1^=3455 (br, O−H), 2923 (m), 1758 (s), 1657 (s), 1619 (s), 1503 (s), 1412 (s), 1310 (s), 1218 (m), 1180 (m), 1133 (m), 1039 (m), 829 (m). ^**1**^ **H NMR** (400 MHz, CDCl_3_) **δ_H_** 8.20 (dd, *J_1_* =*J_2_* =8.3 Hz, 1H), 7.98 (d, *J=*8.2 Hz, 1H), 7.96 (d, *J=*1.5 Hz, 1H), 7.83 (dd, *J*_1_ *=*8.2 Hz, *J*_2_ *=*1.7 Hz, 1H), 7.79 (s, 1H), 7.23 (dd, *J*_1_ *=*8.3 Hz, *J*_2_ *=*1.7 Hz, 1H), 7.17 (dd, *J*_1_ *=*10.6 Hz, *J*_2_ *=*1.7 Hz, 1H), 7.17 (brs, 1H), 7.13 (d, *J=*8.7 Hz, 2H), 7.11 (d, *J=*8.7 Hz, 2H), 6.80 (d, *J=*8.7 Hz, 4H), 4.62 (s, 2H), 4.51 (t, *J=*4.9 Hz, 2H), 4.19 (quintet, *J=*5.3 Hz, 1H), 4.14–4.09 (m, 1H), 4.05 (t, *J=*5.0 Hz, 2H), 3.97 (d, *J=*1.9 Hz, 1H), 3.96 (d, *J=*3.0 Hz, 1H), 3.86 (t, *J=*5.0 Hz, 2H), 3.77 (dd, *J*_1_ *=*11.7 Hz, *J*_2_ *=*5.3 Hz, 1H), 3.77–3.45 (m, 19H), 3.17 (brs, 2H), 1.66–1.60 (m, 4H), 1.63 (s, 6H), 1.62 (s, 6H). ^**13**^ **C‐NMR** (126 MHz, CDCl_3_ ) **δ_C_** 179.8, 174.5, 162.9 (d, *J=*3.0 Hz), 160.3 (d, *J=*251 Hz), 156.4, 156.1, 143.9, 143.4, 141.3 (d, *J=*10.7 Hz), 136.8, 135.3, 133.6 (q, *J=*33.6 Hz), 133.2 (d, *J=*3.3 Hz), 132.2, 127.8, 127.7, 127.1 (q, *J*=4.76 Hz), 126.1 (d, *J*=3.2 Hz), 123.0 (d, *J*=12.6 Hz), 121.8 (q, *J=*274 Hz), 117.9 (d, *J=*26 Hz), 113.9 (d, *J=*1.1 Hz), 113.00×2, 110.4 (q, *J=*2.0 Hz), 71.6, 71.2, 70.50×3, 70.40×5, 69.9, 69.5, 69.3, 69.1, 68.9, 68.4, 66.6, 63.8, 46.0, 41.7, 39.9, 30.9, 26.3×2, 23.8. ^**19**^ **F‐NMR** (376 MHz, CDCl_3_) −62.9, −111.4. **HRMS** (ESI+): m/*z* [M+H]^+^ calculated for C_56_H_67_ClF_4_N_7_O_11_S: **1156.4244**; found **1156.4247**. error: 0.3 ppm. **HPLC purity** 97.2 %.

### Biological characterisation

#### General considerations

EPI‐001 and Epoxomicin were purchased from SelleckChem and Enzalutamide (MDV‐3100) was supplied by ApexBio. AR‐FL‐Gal4 and p(UAS4)‐TATA‐luc plasmids were a gift from Scott Dehm while the Renilla‐luc plasmid was bought commercially (Promega). PCR primers were designed in‐house and synthesised by Merck. Graphs were created and processed using the GraphPad Prism software.

#### Cell lines

C4‐2b, PC‐3 and CV‐1 cells were procured through commercial suppliers. C4‐2b and PC‐3 were cultured in RPMI media while CV‐1 were cultured in EMEM; all with supplementation of 10 % FBS, 1 % L‐glutamine and 1 % Penicillin/Streptomycin. All cells were cultured in a humidified incubator at 37 °C with 5 % CO2.

#### Cell viability assays

AR‐positive C4‐2b cells were treated with compounds at a range of concentrations for 72hr in full media in 96‐well plates. The range of concentrations for C4‐2b were mirrored in AR‐null PC‐3 cells. Cell viability was determined using the CellTiter 96 Aqueous MTS assay (Promega), as per supplier's instructions whereby cells were incubated with the reagent 3‐(4,5‐dimethylthiazol‐2‐yl)‐5‐(3‐carboxymethoxyphenyl)‐2‐(4‐sulfophenyl)‐2H‐tetrazolium) and OD was measured following an incubation period. Viability was calculated by normalising to negative control wells containing media only and displaying as a percentage of DMSO‐treated cells. The DMSO concentration was 0.1 % in all wells.

#### Luciferase assays

CV‐1 cells were transfected with plasmids AR‐FL‐Gal4, p(UAS4)‐TATA‐luc and internal control, Renilla‐luc reporter plasmid using Lipofectamine 2000 (Invitrogen) in a 10 cm dish following manufacturer's protocol. After 24hr, cells were treated with drugs +/− hormone in a white 96‐well plate in medium containing charcoal‐stripped FBS. 48hr post‐treatment, firefly and renilla luciferase signals were measured using the Twinlite kit (Perkin Elmer) according to manufacturer's instructions. Transactivation of the AR was calculated by normalising to the Renilla internal control and was displayed as a fold‐induction compared to Vehicle no drug control wells. The DMSO concentration was 0.1 % in all wells.

#### RT‐qPCR

Cells were treated +/− R1881 and with drug compounds/DMSO for 24 hr in 6‐well plates. Cells were then harvested, and RNA extracted using the RNeasy Plus Mini Kit (Qiagen), according to the kit's instructions. RNA was then reverse transcribed using the High Capacity cDNA Reverse Transcription Kit (Applied Biosystems). For qPCR, the QuantiNova SYBR Green PCR Kit (Qiagen) was used to measure gene expression compared to the internal control gene, GAPDH. Delta‐delta‐ct analysis method was applied to generated values such that conditions were displayed as a fold‐induction compared to Vehicle no drug control wells (containing the equivalent DMSO concentration as drug‐treated wells). The DMSO concentration was 0.1 % in all wells.

## Conflict of interest

The authors declare no conflict of interest.

1

## Supporting information

As a service to our authors and readers, this journal provides supporting information supplied by the authors. Such materials are peer reviewed and may be re‐organized for online delivery, but are not copy‐edited or typeset. Technical support issues arising from supporting information (other than missing files) should be addressed to the authors.

Supporting Information

## Data Availability

All data supporting this study are included in the paper and provided as Supporting Information.
