# Supplementary material for: Hybrid Androgen Receptor Inhibitors Outperform Enzalutamide and EPI‐001 in in vitro Models of Prostate Cancer Drug Resistance
Source: ChemMedChem. 2022 Nov 15;18(2):e202200548. doi: 10.1002/cmdc.202200548 (PMC10098645; doi:10.1002/cmdc.202200548)
Supplement: Supplementary file 1 — Supporting Information [file CMDC-18-0-s001.pdf]

ChemMedChem

Supporting Information

Hybrid Androgen Receptor Inhibitors Outperform Enzalutamide and EPI-001 in *in vitro* Models of Prostate Cancer Drug Resistance

Radu Costin Bizga Nicolescu[+], Zoe R. Maylin[+], Francisco Javier Pérez-Areales, Jessica Iegre, Hardev S. Pandha, Mohammad Asim,* and David R. Spring*

Supplementary information

Table of Contents

Supplementary Figures 1

1.1. Supplementary Figure 1 – Western blot of C4-2b cells showing the presence of AR variants 1

NMR spectra 2

HPLC data 23

Calculated errors for cytotoxicity studies 26

Statisitical significance for luciferase assays and RT-qPCR experiments 27

Supplementary Figures

1.1. Supplementary Figure 1 – Western blot of C4-2b cells showing the presence of AR variants

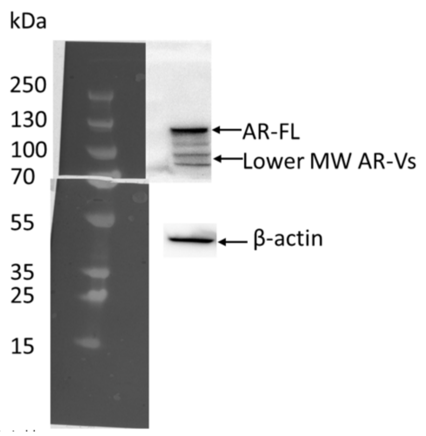

Supplementary figure 1. Western blots of C4-2b cells showing the presence of both AR-FL and AR-Vs

NMR spectra

2

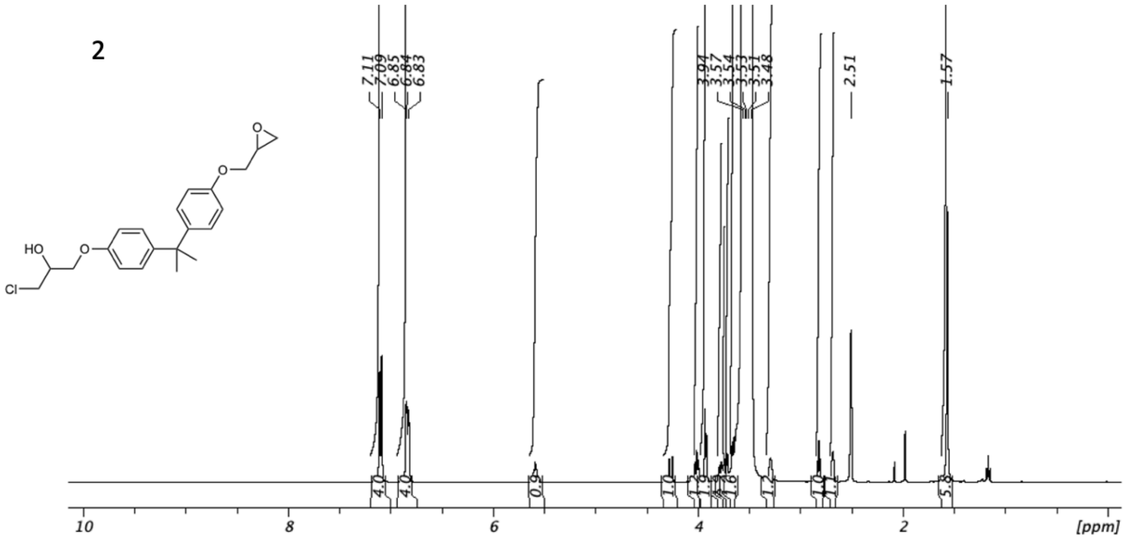

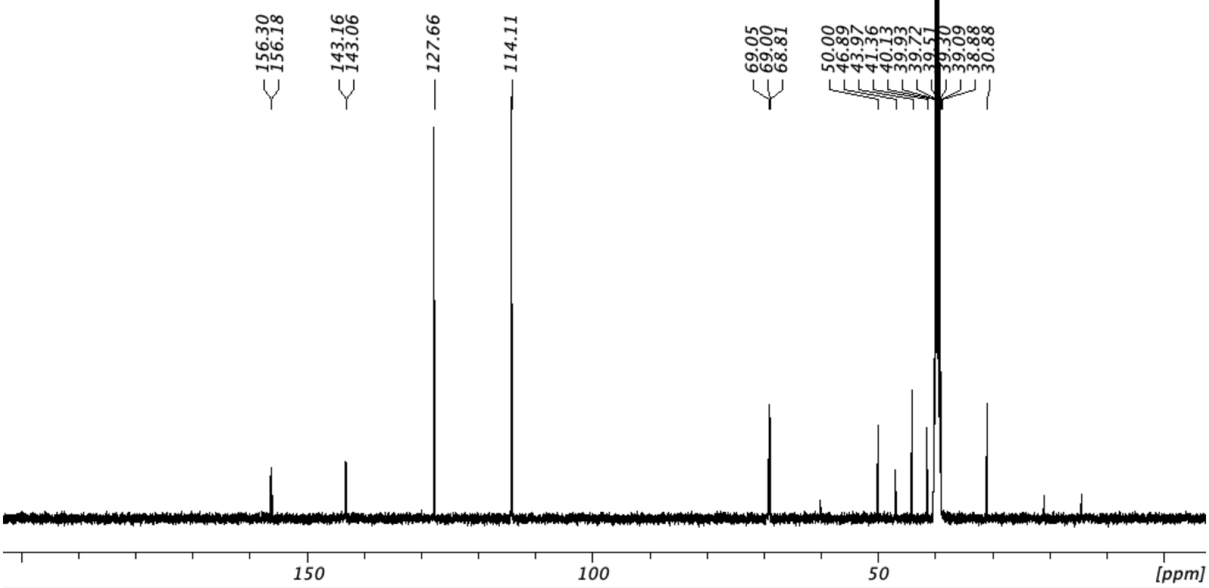

3a

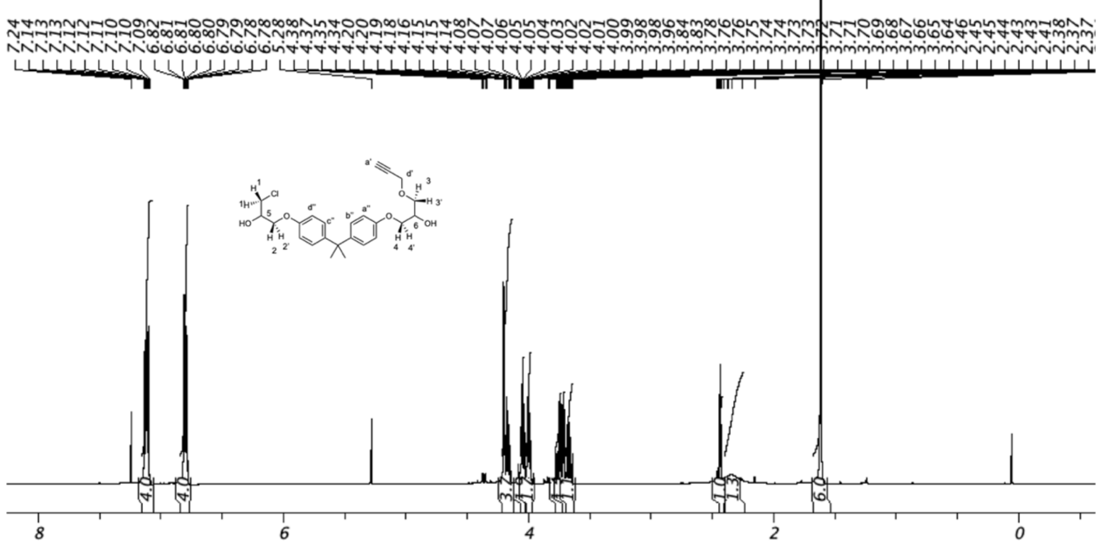

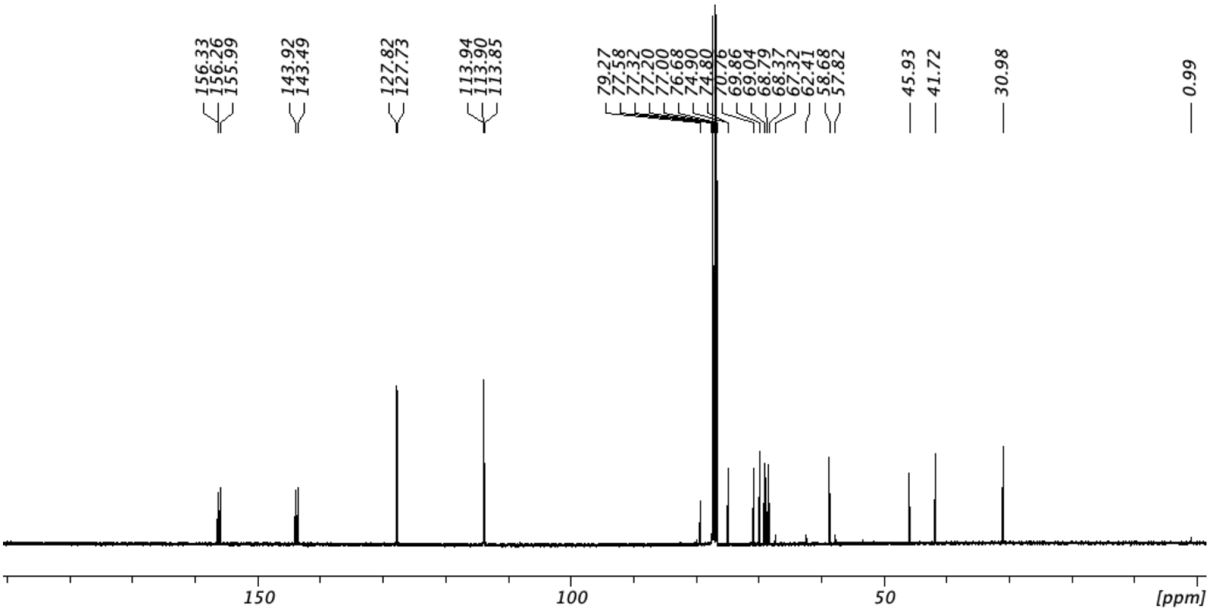

3b

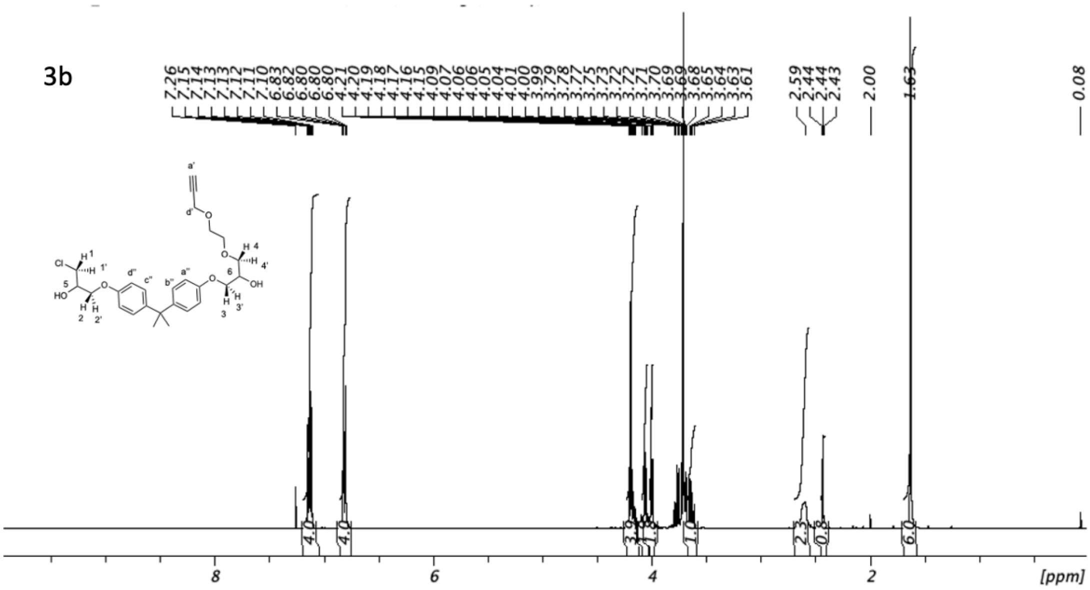

7.26 7.15 7.14 7.13 7.13 7.12 7.11 7.10 6.83 6.82 6.80 6.80 6.80 4.21 4.20 4.19 4.18 4.17 4.16 4.15 4.09 4.07 4.06 4.05 4.04 4.01 4.00 3.99 3.79 3.78 3.77 3.75 3.73 3.72 3.72 3.71 3.70 3.69 3.68 3.65 3.64 3.63 3.61
2.59 2.44 2.44 2.43
2.00
1.63
0.08
4.0
4.0
3.9
1.0
2.3
0.8
6.0
8
6
4
2
[ppm]

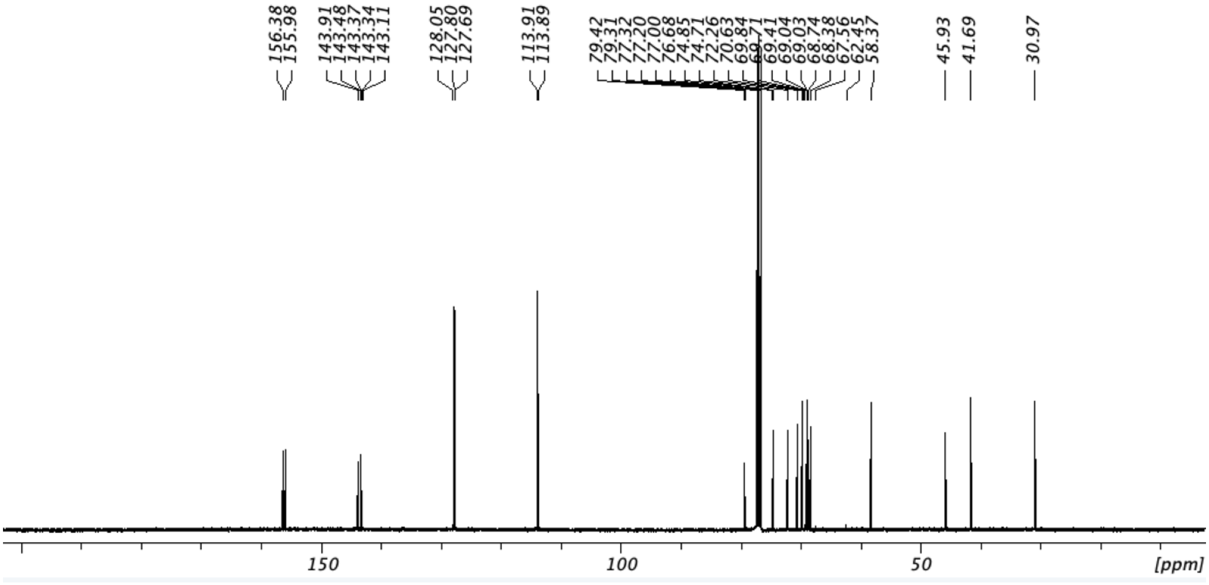

156.38 155.98
143.91 143.48 143.37 143.34 143.11
128.05 127.80 127.69
113.91 113.89
79.42 79.31 77.32 77.20 77.00 76.68 74.85 74.71 72.26 70.63 69.84 69.71 69.41 69.04 69.03 68.74 68.38 67.56 62.45 58.37
45.93
41.69
30.97
150
100
50
[ppm]

3c

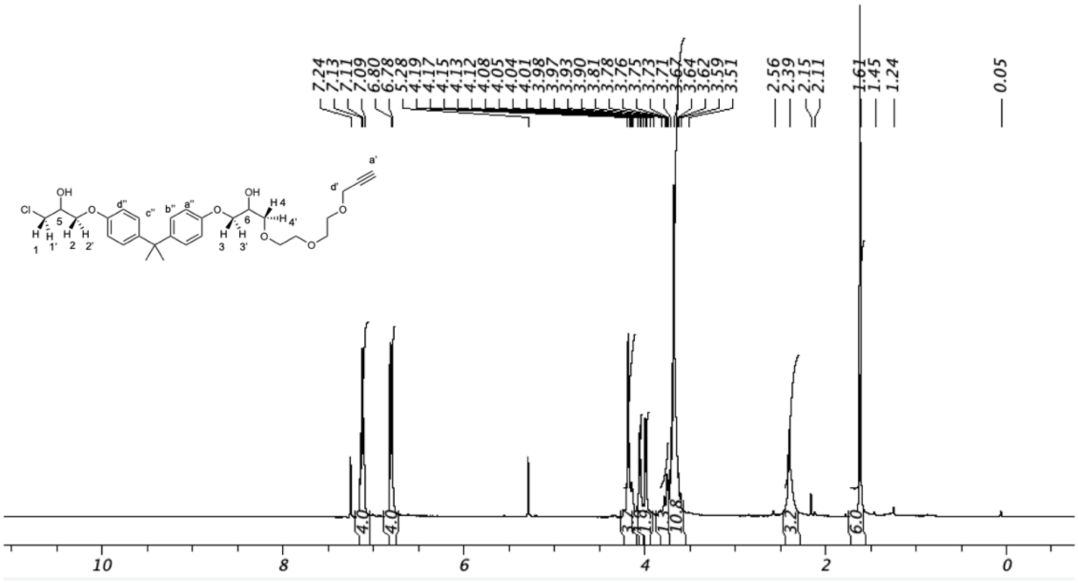

7.24 7.13 7.11 7.09 6.80 6.78 5.28 4.19 4.17 4.15 4.13 4.12 4.08 4.05 4.04 4.01 3.98 3.97 3.93 3.90 3.81 3.78 3.76 3.75 3.73 3.71 3.67 3.64 3.62 3.59 3.51 2.56 2.39 2.15 2.11 1.61 1.45 1.24 0.05
Cl
OH
d''
c''
b''
a''
H 4
H 4'
d'
a'
1 1' 2 2'
5
6
3 3'
4.0
4.0
10.8
3.2
6.0
10
8
6
4
2
0

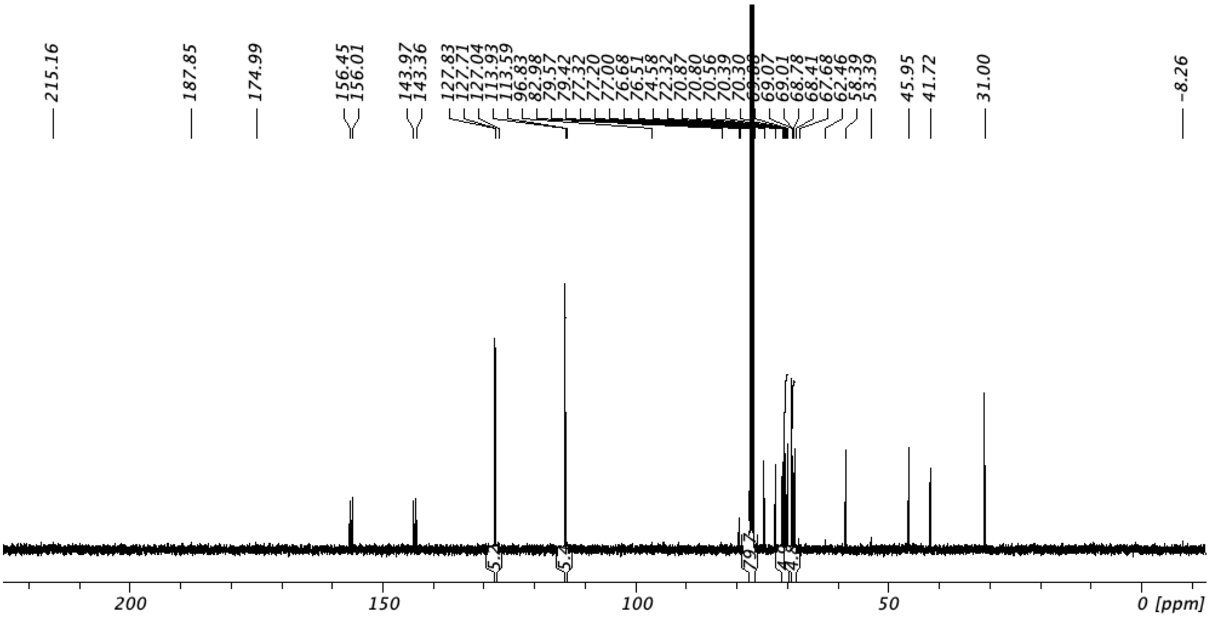

215.16 187.85 174.99 156.45 156.01 143.97 143.36 127.83 127.71 127.04 113.93 113.59 96.83 82.98 79.57 79.42 77.32 77.20 77.00 76.68 76.51 74.58 72.32 70.87 70.80 70.56 70.39 70.30 69.07 69.01 68.78 68.41 67.68 62.46 58.39 53.39 45.95 41.72 31.00 -8.26
200
150
100
50
0 [ppm]

3d

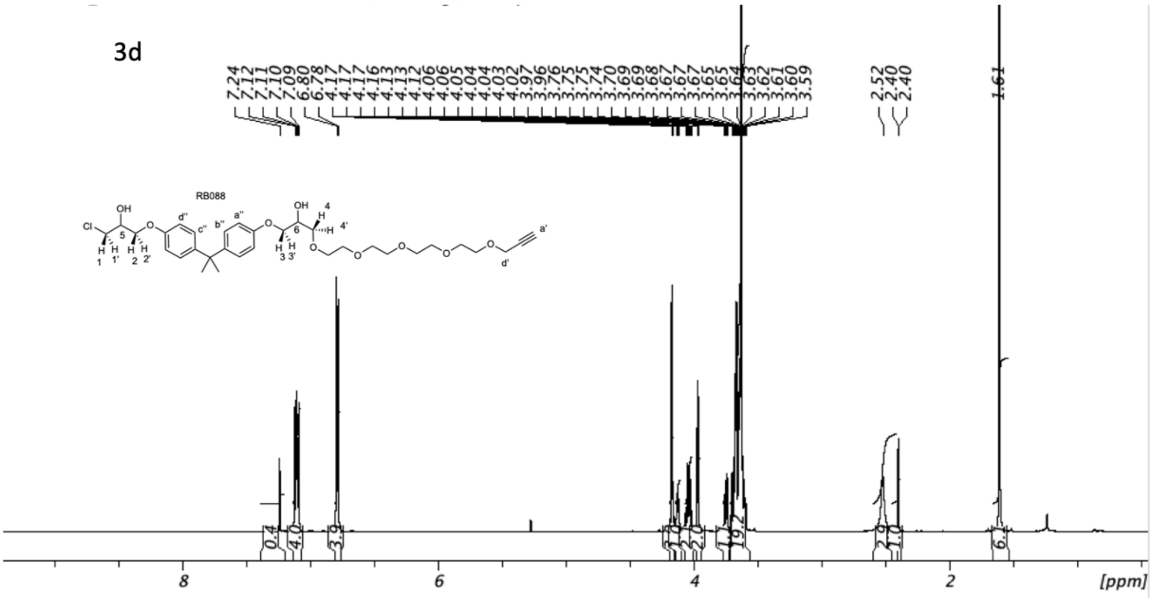
7.24
7.12
7.11
7.10
7.09
6.80
6.78
4.17
4.17
4.16
4.13
4.13
4.12
4.06
4.05
4.04
4.04
4.03
4.02
3.97
3.96
3.76
3.75
3.74
3.70
3.69
3.68
3.67
3.67
3.65
3.64
3.63
3.62
3.61
3.60
3.59
2.52
2.40
2.40
1.61
RB088
0.4
4.0
3.9
2.0
19.2
1.0
8
6
4
2
[ppm]

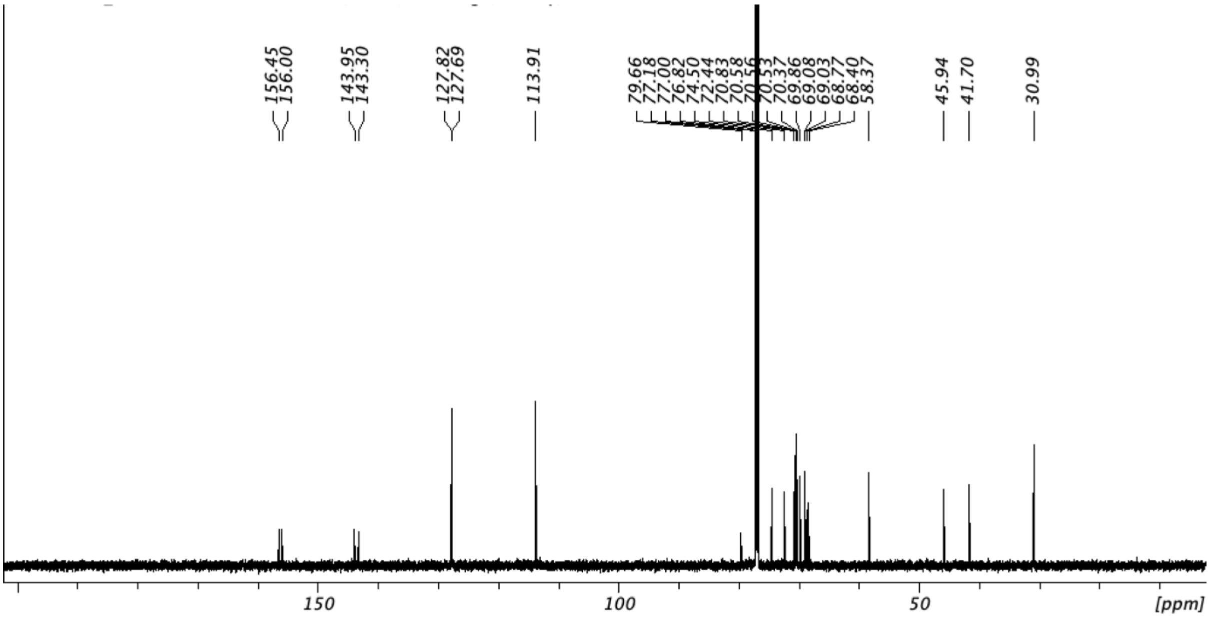
156.45
156.00
143.95
143.30
127.82
127.69
113.91
79.66
77.18
77.00
76.82
74.50
72.44
70.83
70.58
70.56
70.53
70.37
69.86
69.08
69.03
68.77
68.40
58.37
45.94
41.70
30.99
150
100
50
[ppm]

3e

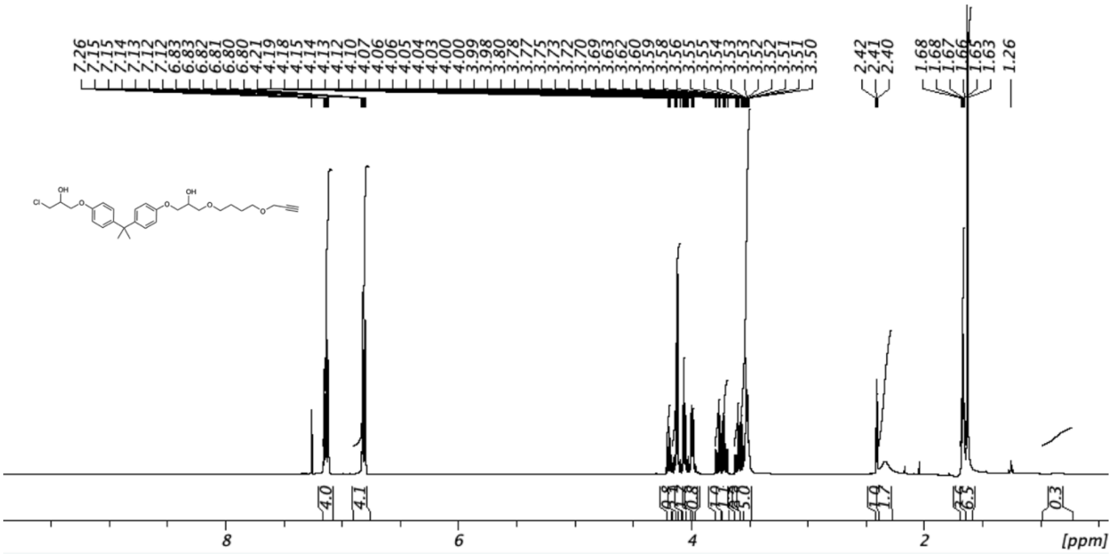

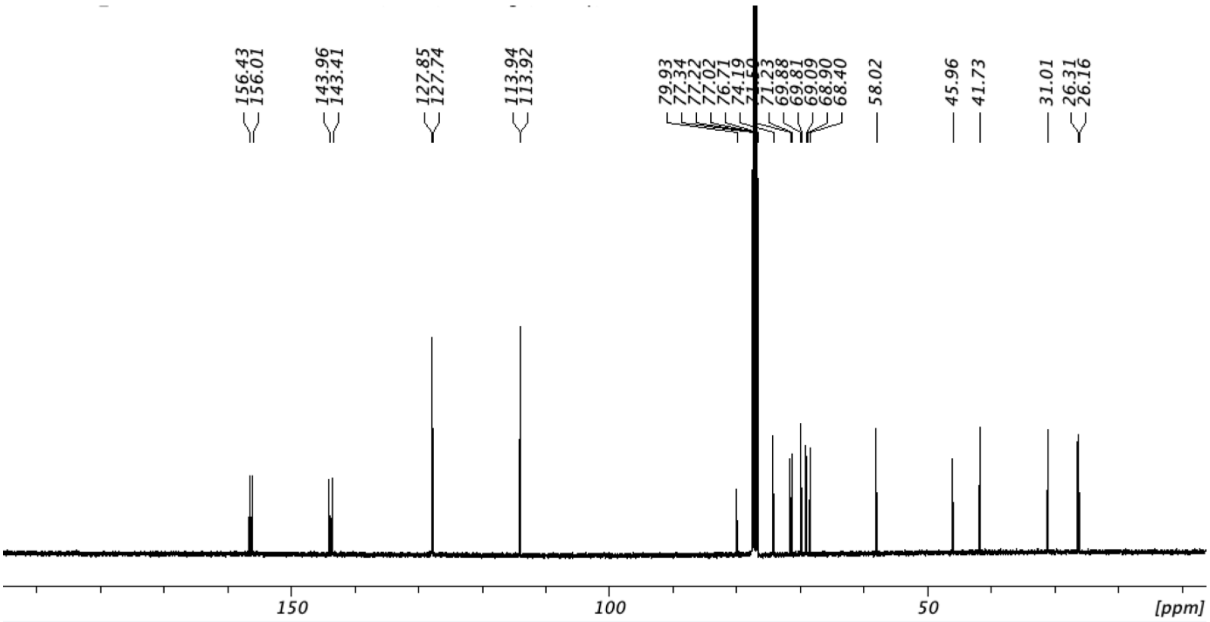

5b

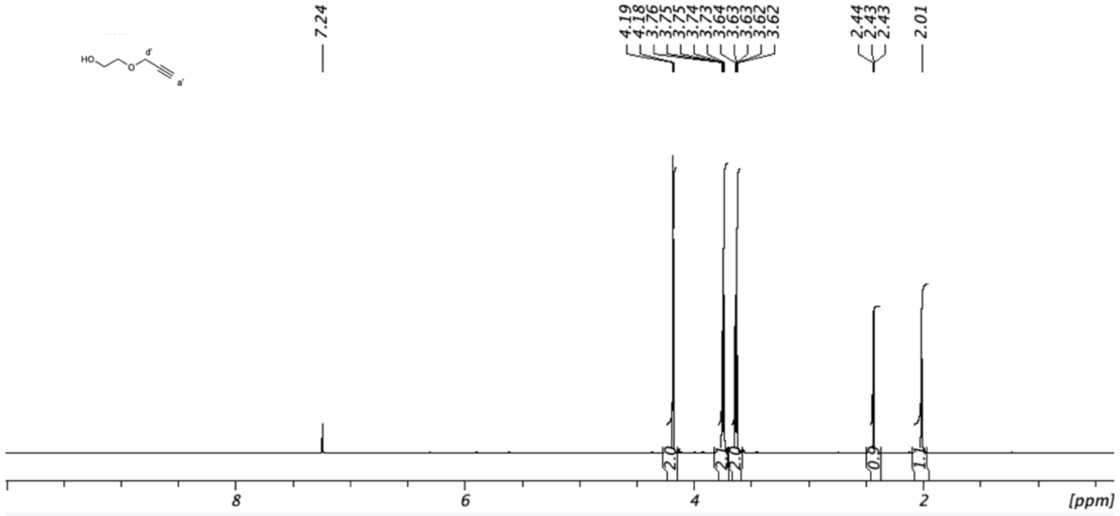
HO
O
d'
a'
7.24
4.19
4.18
3.76
3.75
3.75
3.74
3.73
3.64
3.63
3.63
3.62
3.62
2.44
2.43
2.43
2.01
2.0
2.1
2.0
0.9
1.1
8
6
4
2
[ppm]

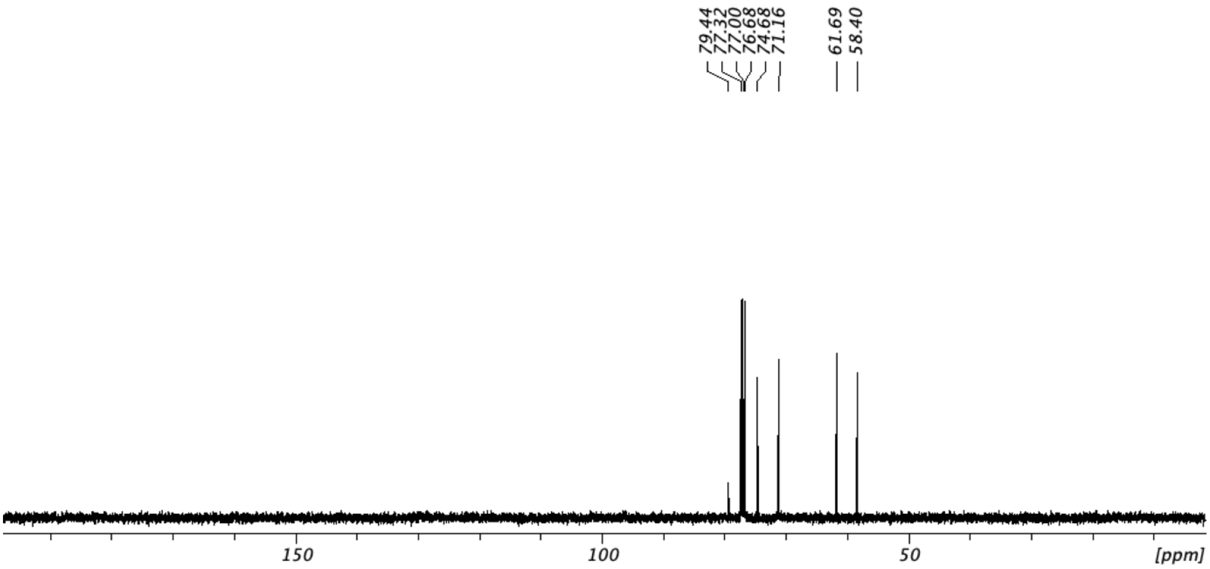
79.44
77.32
77.00
76.68
74.68
71.16
61.69
58.40
150
100
50
[ppm]

5c

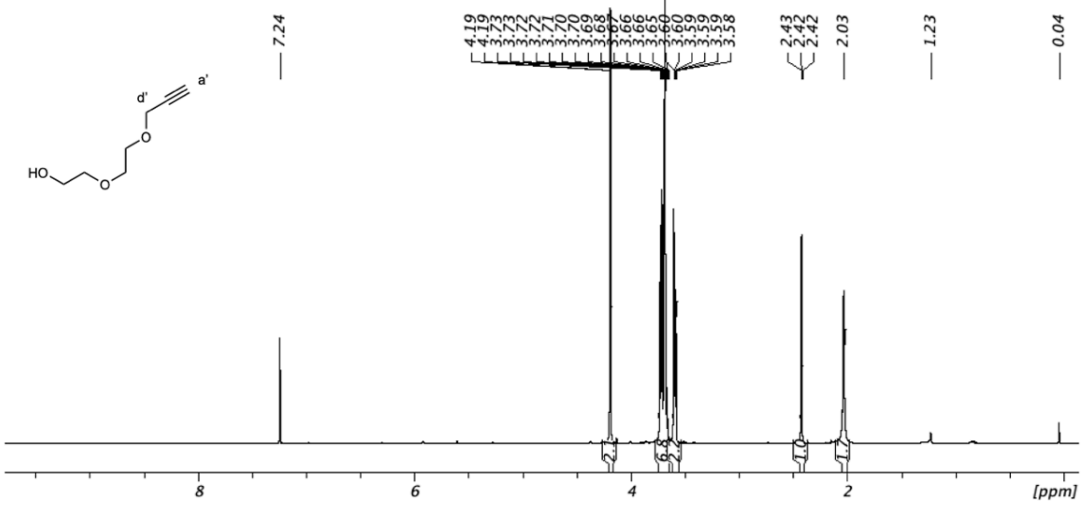
HO
O
O
d'
a'
7.24
4.19
4.19
3.73
3.73
3.72
3.72
3.71
3.70
3.69
3.68
3.67
3.66
3.66
3.65
3.60
3.60
3.59
3.59
3.59
3.58
2.43
2.42
2.42
2.03
1.23
0.04
8
6
4
2
[ppm]

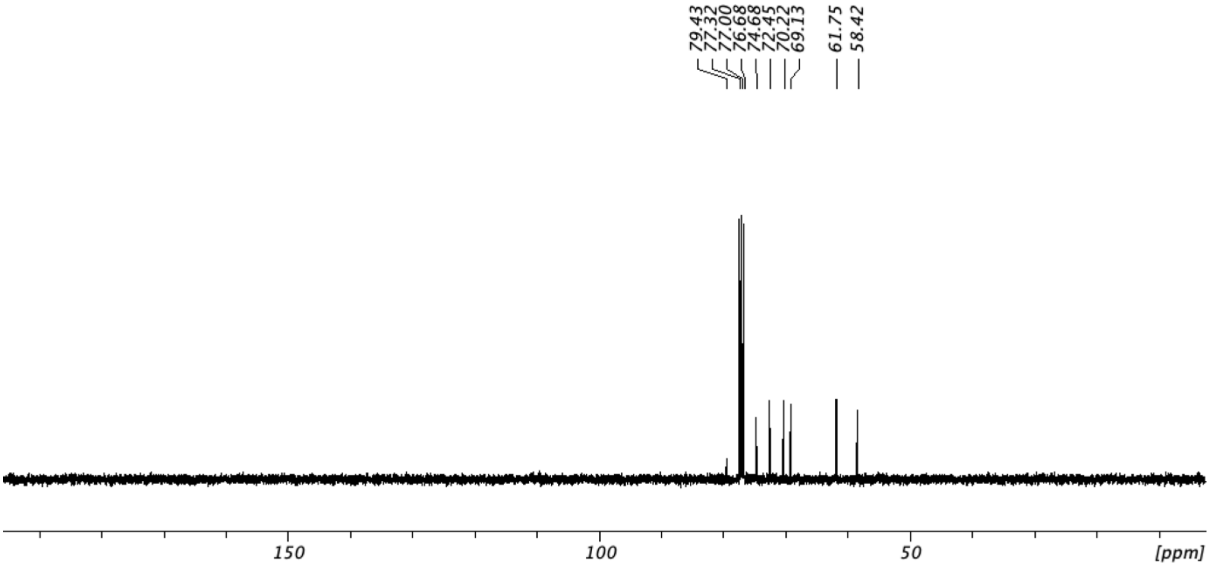
79.43
77.32
77.00
76.68
74.68
72.45
70.22
69.13
61.75
58.42
150
100
50
[ppm]

5d

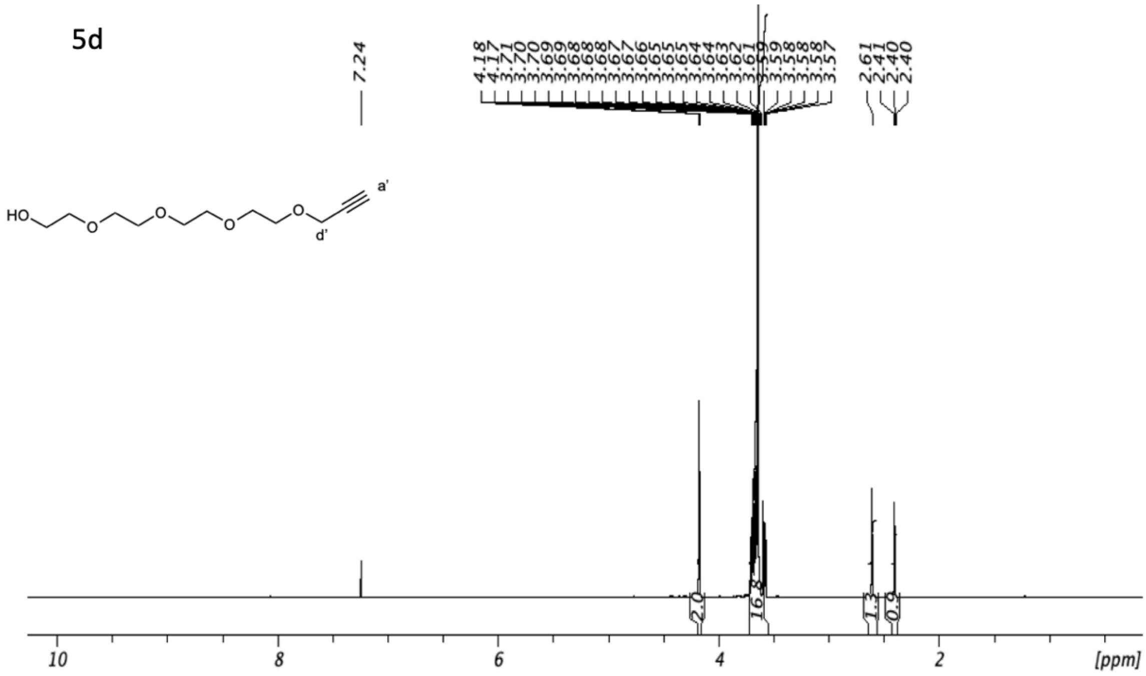
7.24
4.18
4.17
3.71
3.70
3.69
3.69
3.68
3.68
3.68
3.67
3.67
3.66
3.65
3.65
3.65
3.64
3.64
3.63
3.62
3.61
3.59
3.58
3.58
3.58
3.57
2.61
2.41
2.40
2.40
HO
O
O
O
O
a'
d'
2.0
16.8
1.3
0.9
10
8
6
4
2
[ppm]

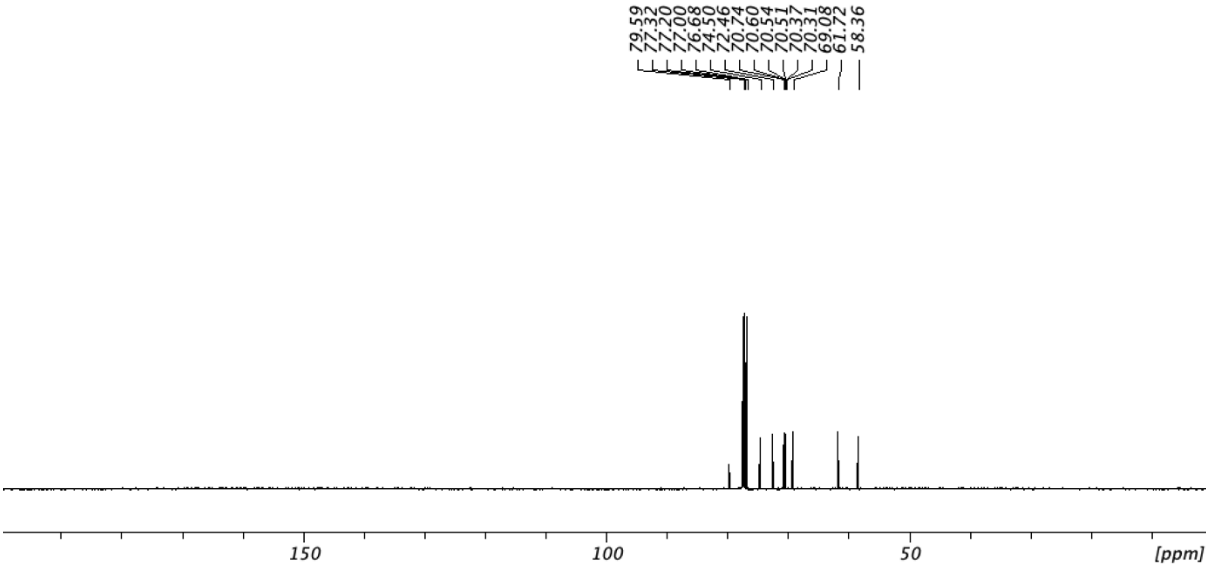
79.59
77.32
77.20
77.00
76.68
74.50
72.46
70.74
70.60
70.54
70.51
70.37
70.31
69.08
61.72
58.36
150
100
50
[ppm]

5e

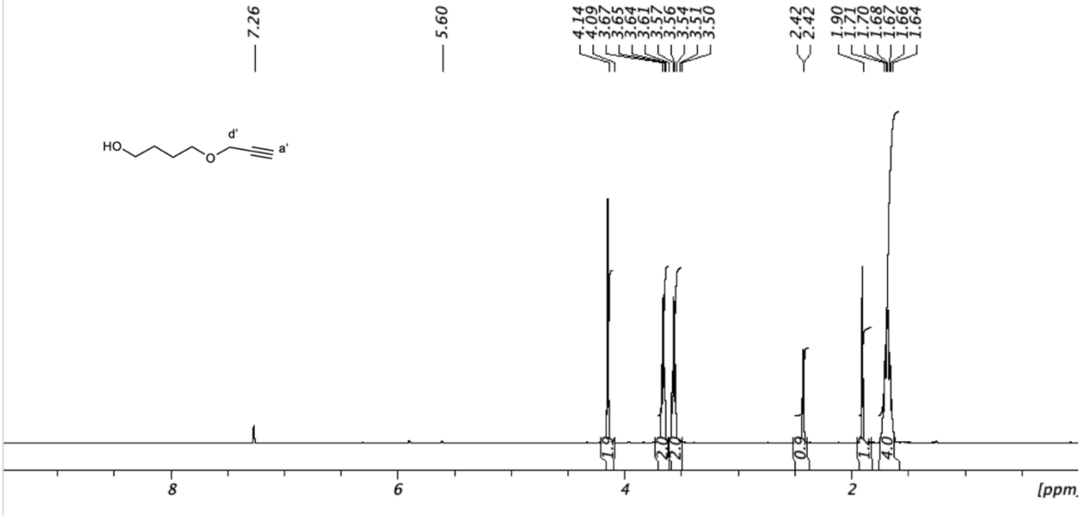
7.26
5.60
4.14 4.09 3.67 3.65 3.64 3.61 3.57 3.56 3.54 3.51 3.50
2.42 2.42
1.90 1.71 1.70 1.68 1.67 1.66 1.64
HO
O
d'
a'
8
6
4
2
[ppm

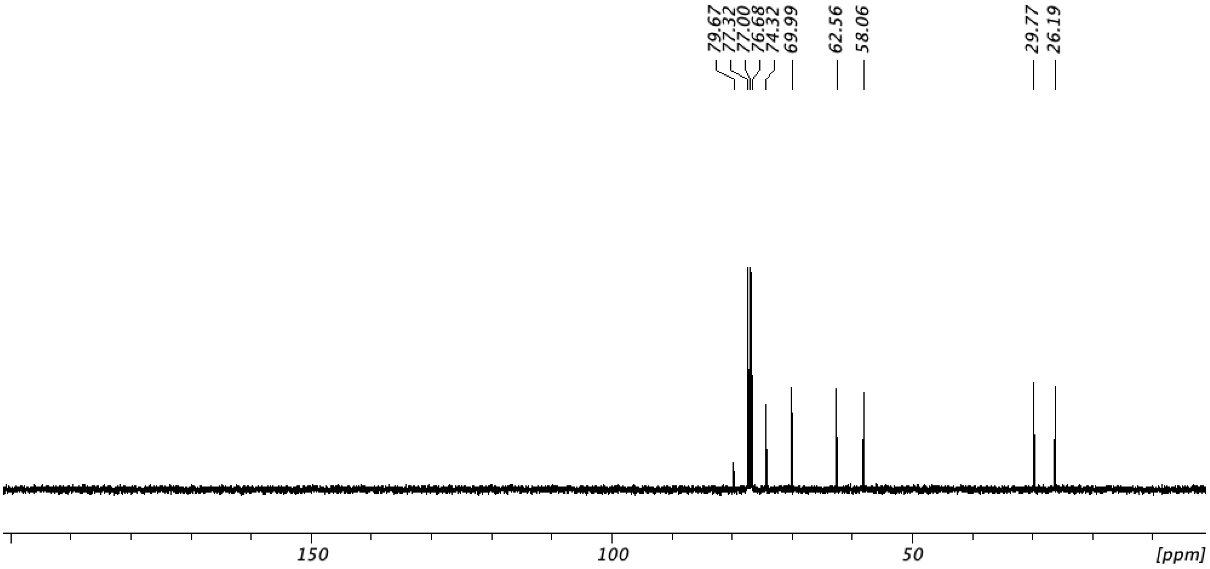
79.67 77.32 77.00 76.68 74.32 69.99
62.56 58.06
29.77 26.19
150
100
50
[ppm]

7

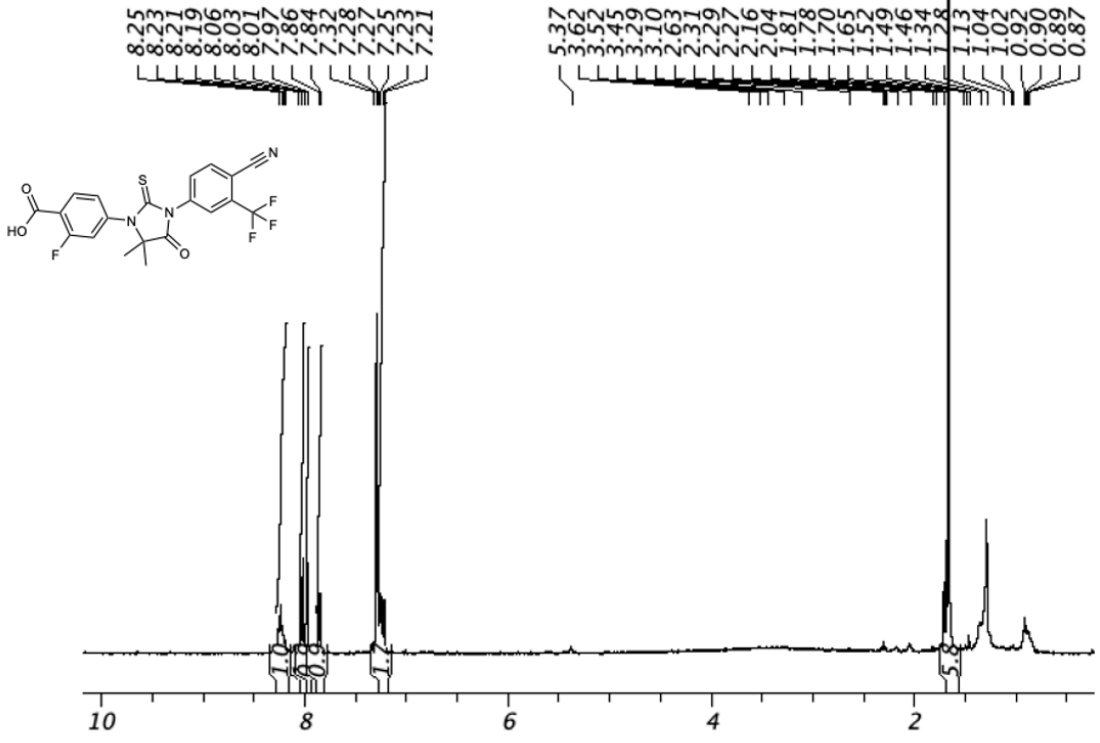
8.25
8.23
8.21
8.19
8.06
8.03
8.01
7.97
7.86
7.84
7.32
7.28
7.27
7.25
7.23
7.21
5.37
3.62
3.52
3.45
3.29
3.10
2.63
2.31
2.29
2.27
2.16
2.04
1.81
1.78
1.70
1.65
1.52
1.49
1.46
1.34
1.28
1.13
1.04
1.02
0.92
0.90
0.89
0.87
O
HO
F
S
N
N
O
N
F
F
F
10
8
6
4
2

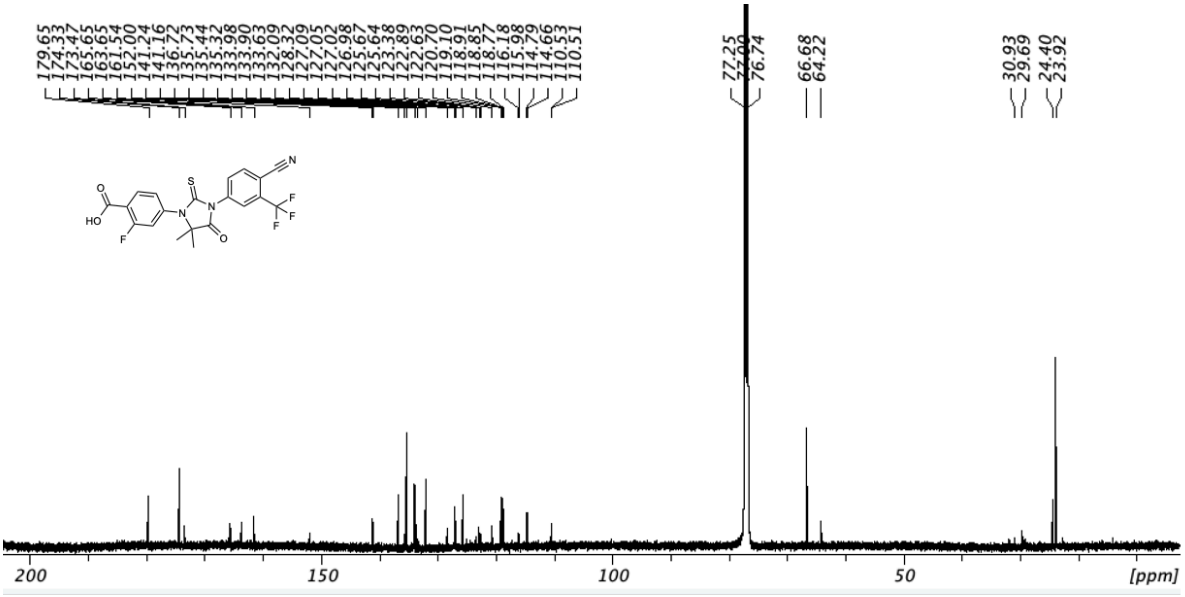
179.65
174.33
173.47
165.65
163.65
161.54
152.00
141.24
141.16
136.72
135.73
135.44
135.32
133.98
133.90
133.63
132.09
128.32
127.09
127.05
127.02
126.98
125.67
125.64
123.38
122.89
122.63
120.70
119.10
118.91
118.85
118.77
116.18
115.98
114.79
114.66
110.53
110.51
77.25
77.00
76.74
66.68
64.22
30.93
29.69
24.40
23.92
O
HO
F
S
N
N
O
N
F
F
F
200
150
100
50
[ppm]

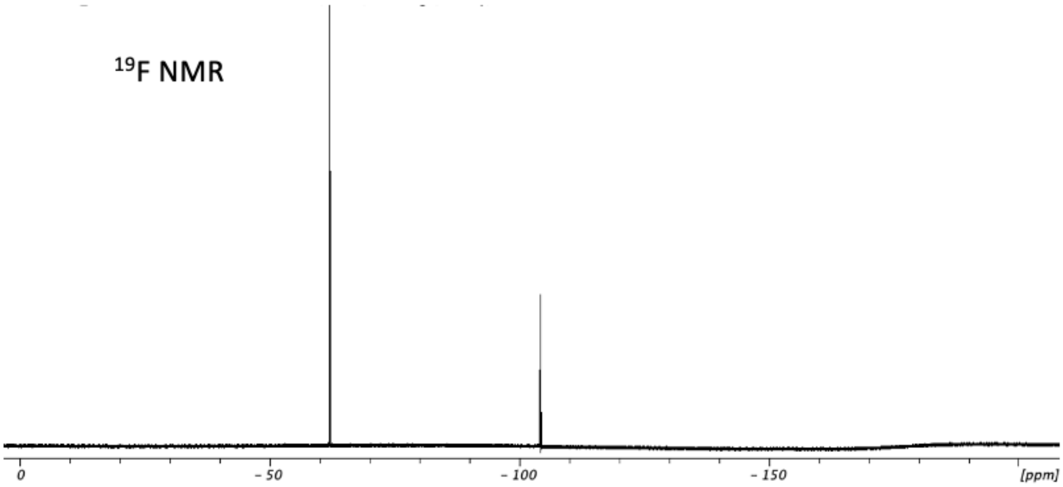

19F NMR
0
- 50
- 100
- 150
[ppm]

8

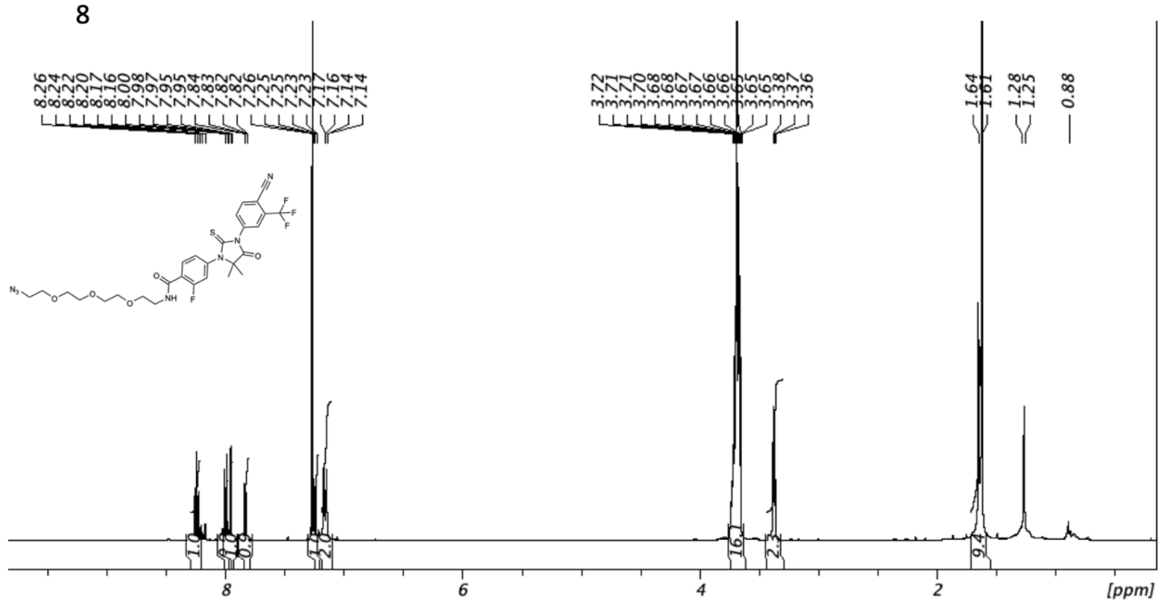
8.26 8.24 8.22 8.20 8.17 8.16 8.00 7.98 7.97 7.95 7.95 7.84 7.83 7.82 7.82 7.26 7.25 7.25 7.23 7.23 7.17 7.16 7.14 7.14
3.72 3.71 3.71 3.70 3.68 3.68 3.67 3.66 3.66 3.65 3.65 3.65 3.38 3.37 3.36
1.64 1.61 1.28 1.25 0.88
N3
O
NH
F
N
S
CF3
16.1
2.3
9.4
8
6
4
2
[ppm]

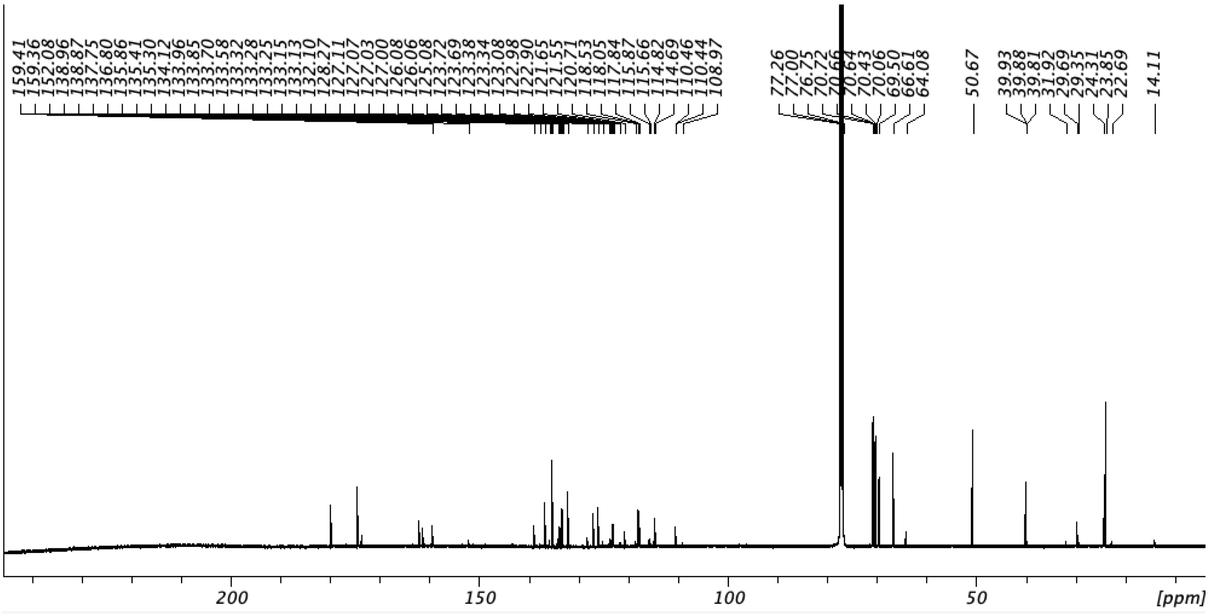
159.41 159.36 152.08 138.96 138.87 137.75 136.80 135.86 135.41 135.30 134.12 133.96 133.85 133.70 133.58 133.32 133.28 133.25 133.15 133.13 132.10 128.27 127.11 127.07 127.03 127.00 126.08 126.06 125.08 123.72 123.69 123.38 123.34 123.08 122.98 122.90 121.65 121.55 120.71 118.53 118.05 115.87 115.84 115.66 114.82 114.69 110.46 110.44 108.97
77.26 77.00 76.75 70.72 70.66 70.64 70.43 70.06 69.50 66.61 64.08
50.67
39.93 39.88 39.81 31.92 29.69 29.35 24.31 23.85 22.69
14.11
200
150
100
50
[ppm]

^{19}F NMR

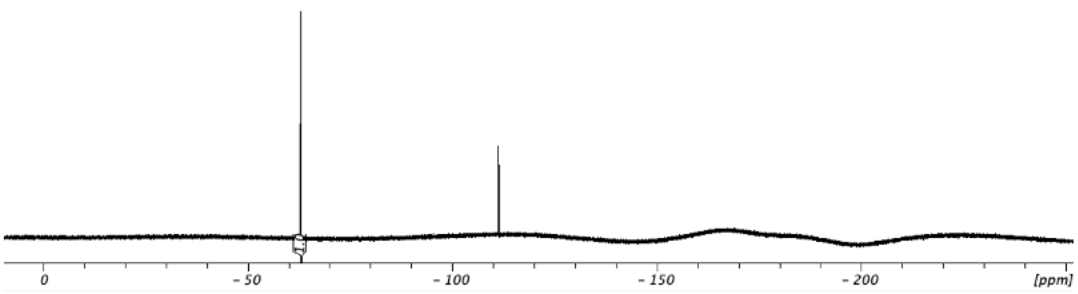

9a

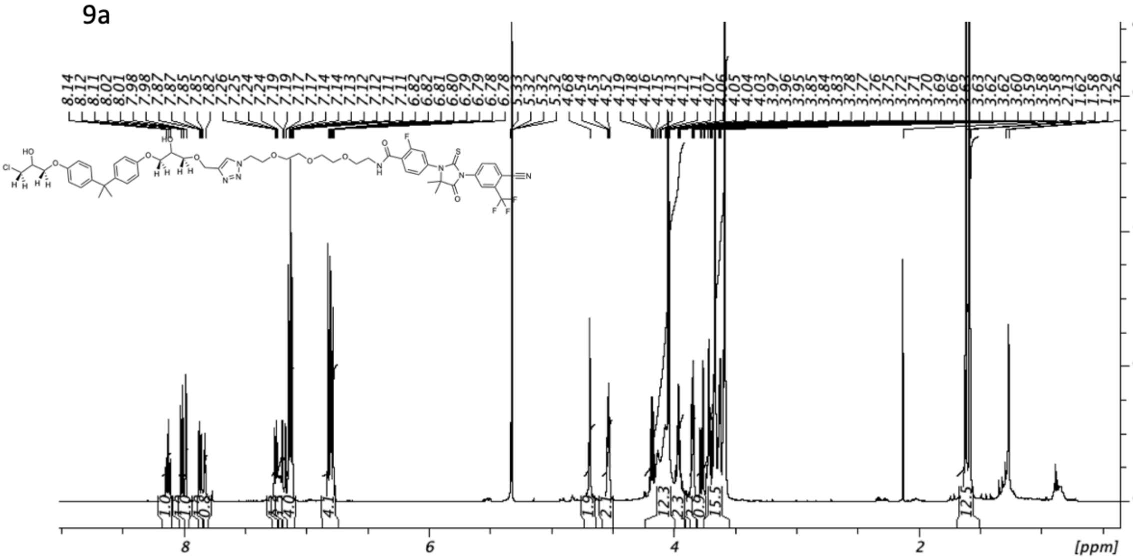

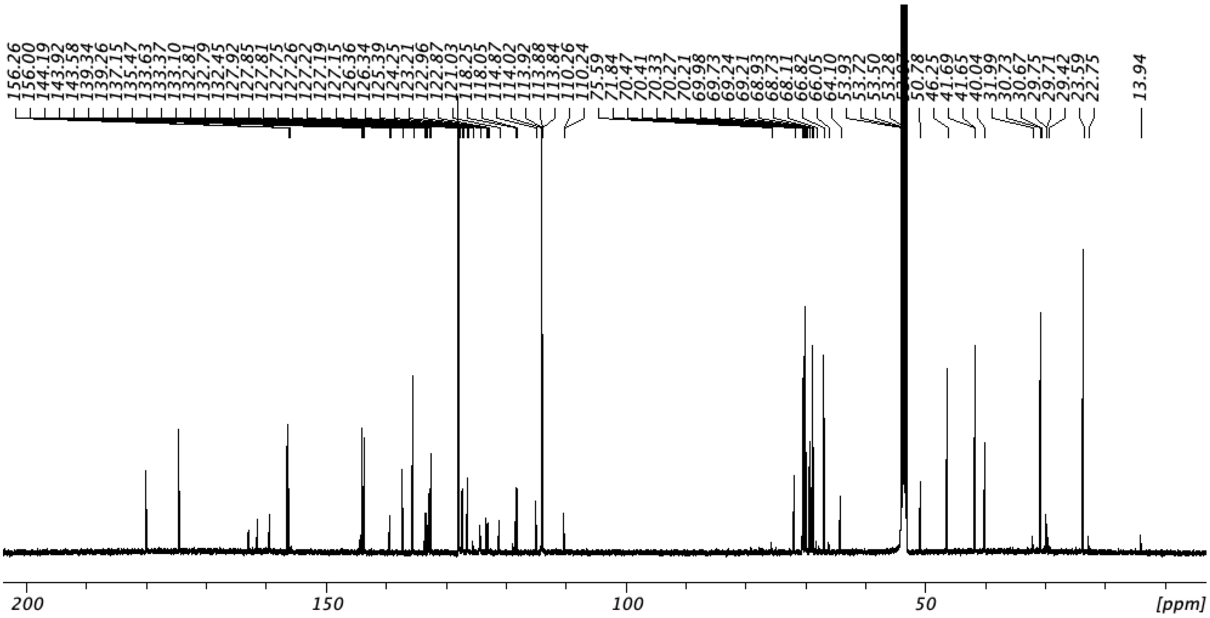

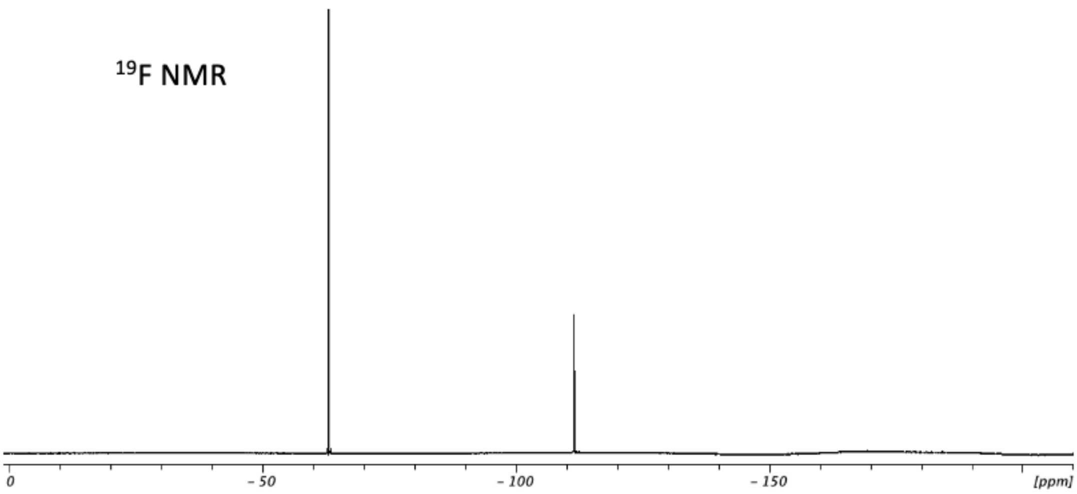

^{19}F NMR
0
– 50
– 100
– 150
[ppm]

9b

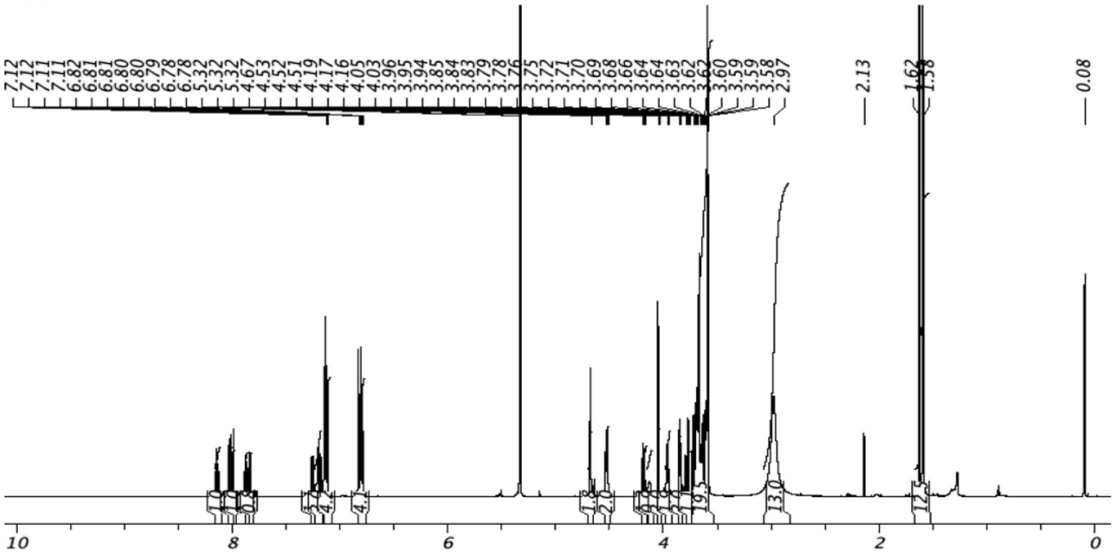
7.12 7.12 7.11 7.11 6.82 6.81 6.81 6.80 6.80 6.79 6.78 6.78 5.32 5.32 5.32 4.67 4.53 4.52 4.51 4.19 4.17 4.16 4.05 4.03 3.96 3.95 3.94 3.85 3.84 3.83 3.79 3.78 3.76 3.75 3.72 3.71 3.70 3.69 3.68 3.66 3.64 3.64 3.63 3.62 3.62 3.60 3.59 3.59 3.58 2.97
2.13
1.62 1.58
0.08
10
8
6
4
2
0

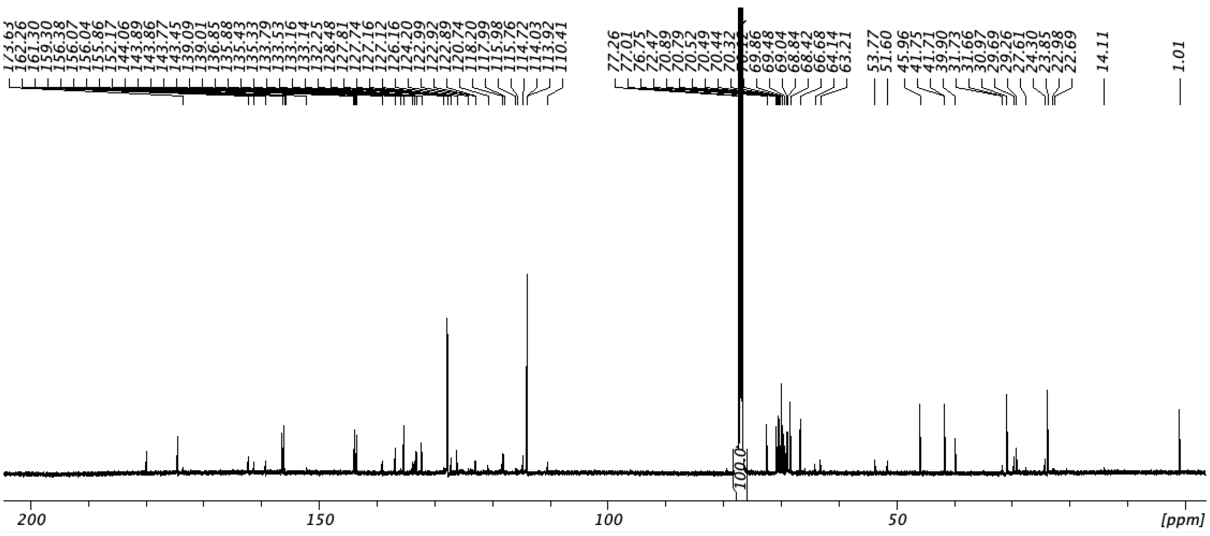
173.63 162.26 161.30 159.30 156.38 156.04 155.86 152.17 144.06 143.89 143.86 143.77 143.45 139.09 139.01 136.85 135.88 135.43 135.33 133.79 133.53 133.16 133.14 132.25 128.48 127.81 127.74 127.16 127.12 126.16 124.20 122.99 122.92 122.89 120.74 118.20 117.99 115.98 115.76 114.72 114.03 113.92 110.41
77.26 77.01 76.75 72.47 70.89 70.79 70.52 70.49 70.44 70.32 69.86 69.48 69.04 68.84 68.42 66.68 64.14 63.21
53.77 51.60 45.96 41.75 41.71 39.90 31.73 31.66 30.97 29.69 29.26 27.61 24.30 23.85 22.98 22.69
14.11
1.01
100.0
200
150
100
50
[ppm]

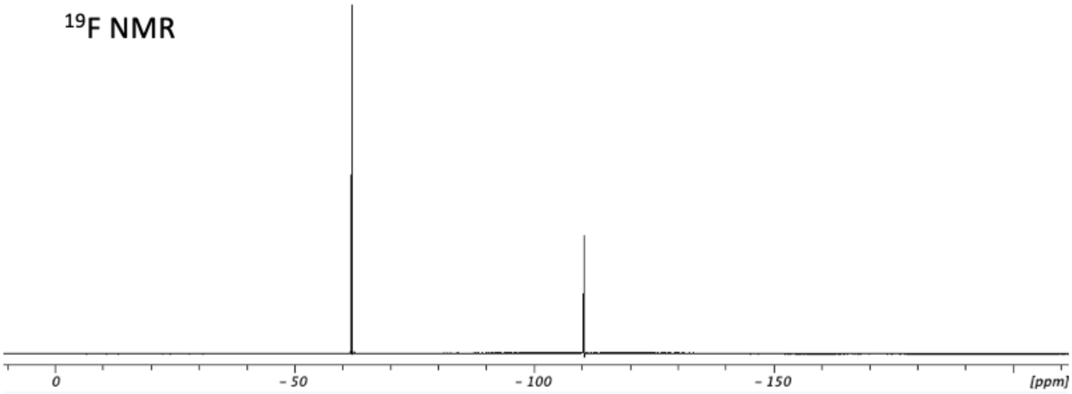

9c

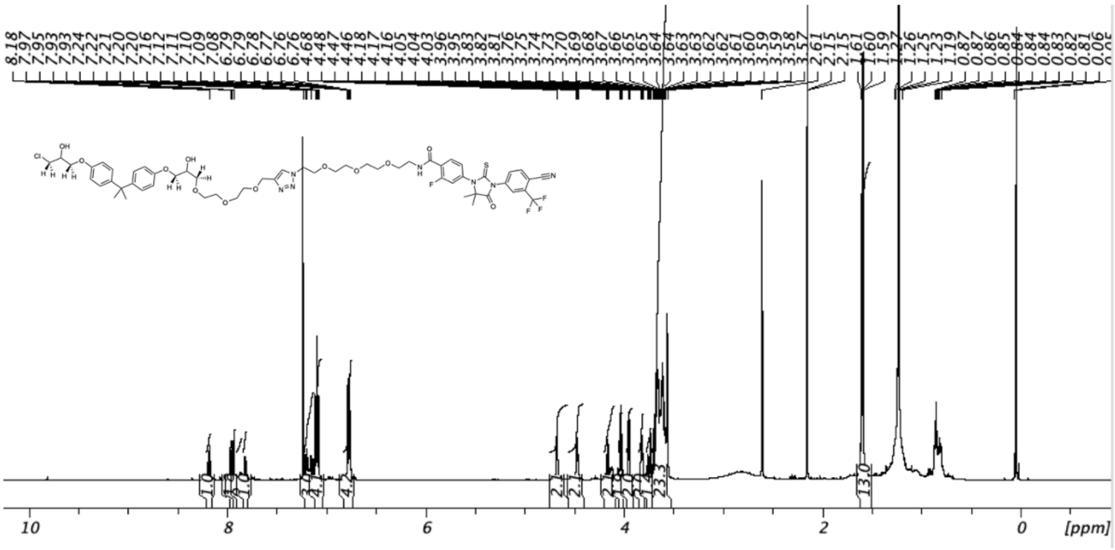

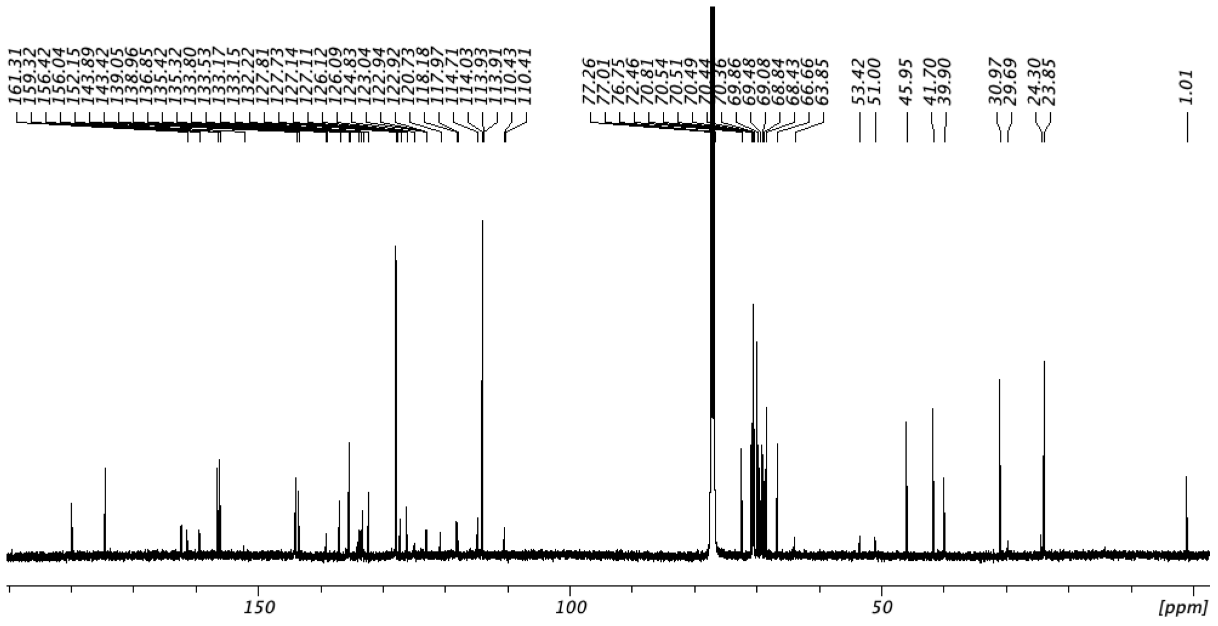

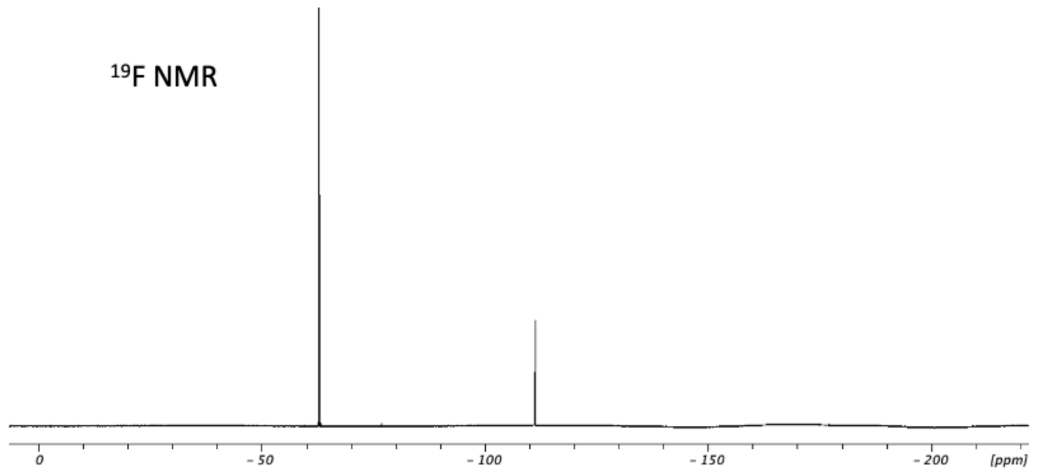
19F NMR

9d

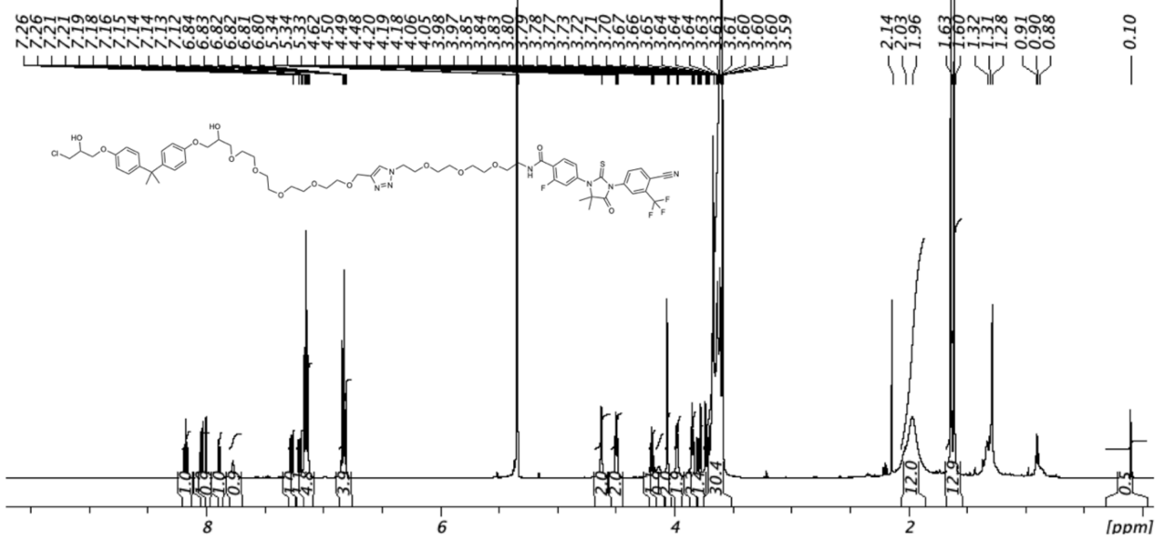
7.26 7.26 7.21 7.21 7.19 7.18 7.16 7.15 7.14 7.14 7.13 7.12 6.84 6.83 6.82 6.82 6.81 6.80 5.34 5.33 5.33 4.62 4.50 4.49 4.48 4.20 4.19 4.18 4.06 4.05 3.98 3.97 3.85 3.84 3.83 3.80 3.79 3.78 3.77 3.73 3.72 3.71 3.70 3.67 3.66 3.65 3.64 3.64 3.64 3.63 3.63 3.61 3.60 3.60 3.60 3.59
2.14 2.03 1.96 1.63 1.60 1.32 1.31 1.28 0.91 0.90 0.88
0.10
8
6
4
2
[ppm]

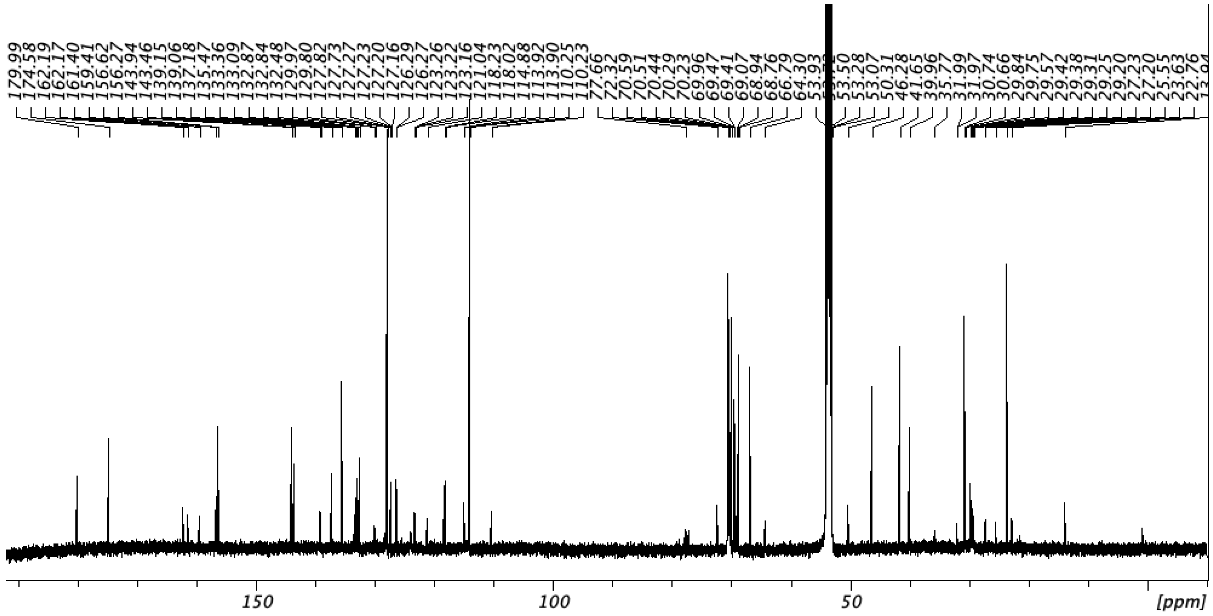
179.99 174.58 162.19 162.17 161.40 159.41 156.62 156.27 143.94 143.46 139.15 139.06 137.47 137.18 135.36 133.09 132.87 132.84 132.48 129.97 129.80 127.93 127.82 127.73 127.27 127.23 127.20 127.16 126.29 126.27 123.26 123.16 121.04 118.23 118.02 114.88 113.92 113.90 110.25 110.23
77.66 72.32 70.59 70.51 70.44 70.29 70.23 69.96 69.47 69.41 69.07 68.94 68.76 66.79 64.30 53.92
53.50 53.28 53.07 50.31 46.28 41.65 39.96 35.77 31.99 31.97 30.74 30.66 29.84 29.75 29.57 29.42 29.38 29.31 29.20 27.23 27.20 25.55 23.63 22.76
150
100
50
[ppm]

^{19}F NMR

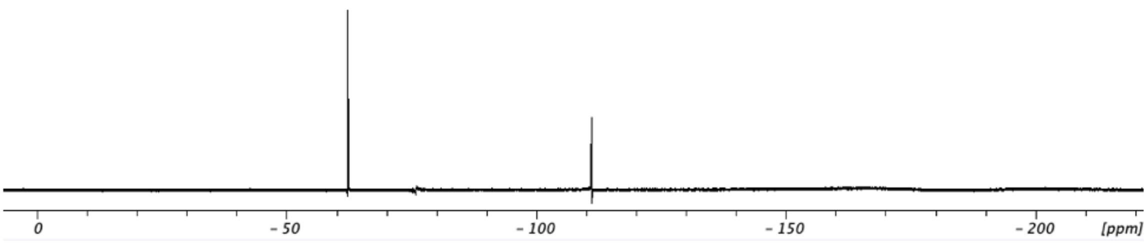

9e

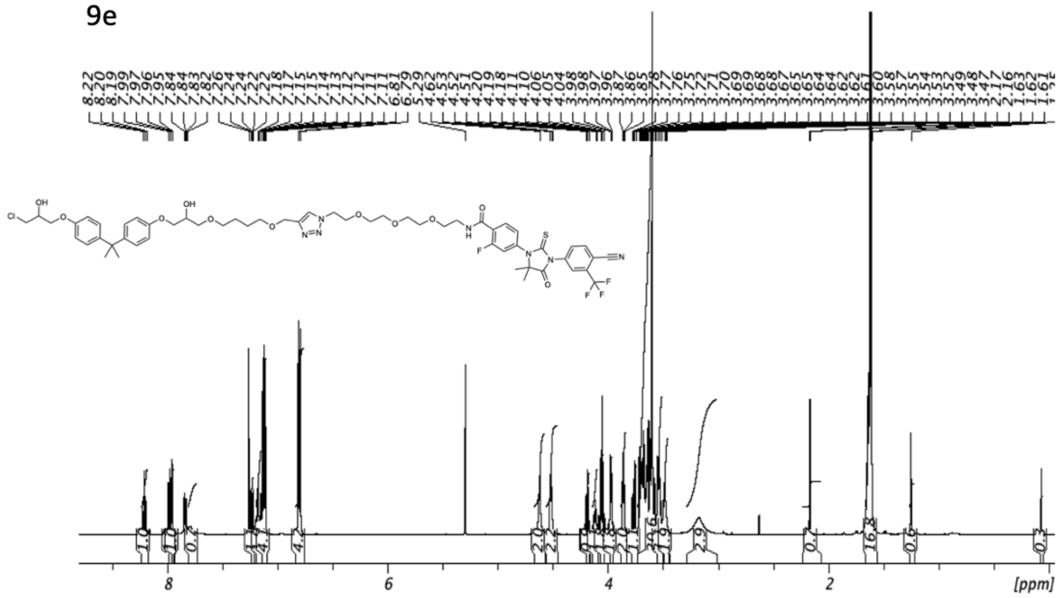

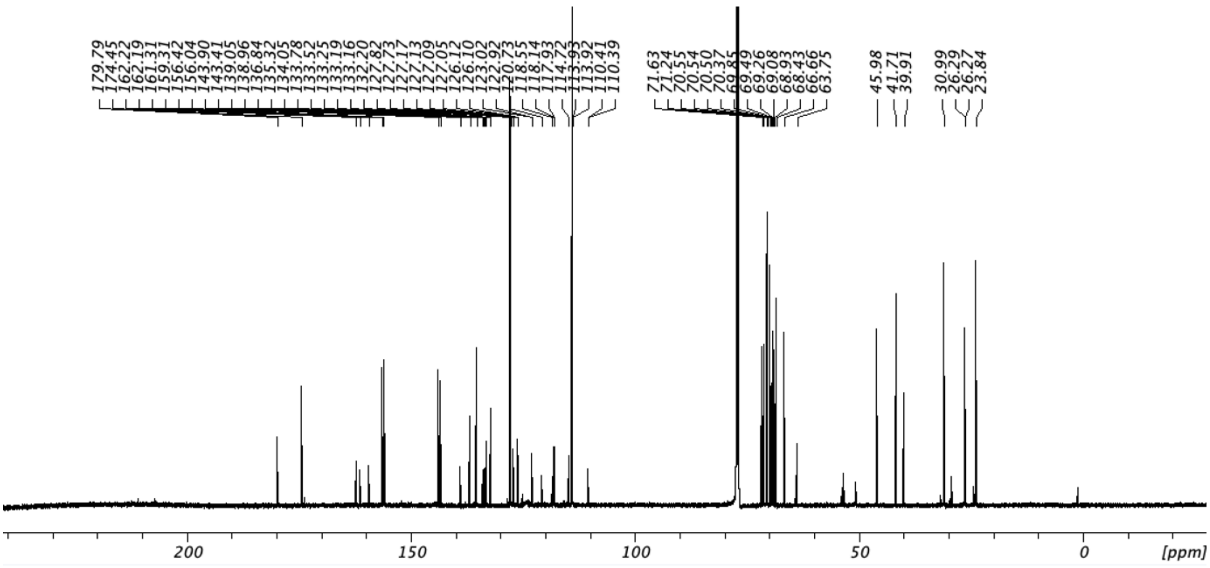
179.79
174.45
162.22
162.19
161.31
159.31
156.42
156.04
143.90
143.41
139.05
138.96
136.84
135.32
134.05
133.78
133.52
133.25
133.19
133.16
132.20
127.82
127.73
127.17
127.13
127.09
127.05
126.12
126.10
123.02
122.92
120.73
118.55
118.14
117.93
114.72
113.93
113.92
110.41
110.39
71.63
71.24
70.55
70.54
70.50
70.37
69.85
69.49
69.26
69.08
68.93
68.43
66.66
63.75
45.98
41.71
39.91
30.99
26.29
26.27
23.84
200
150
100
50
0
[ppm]

^{19}F NMR

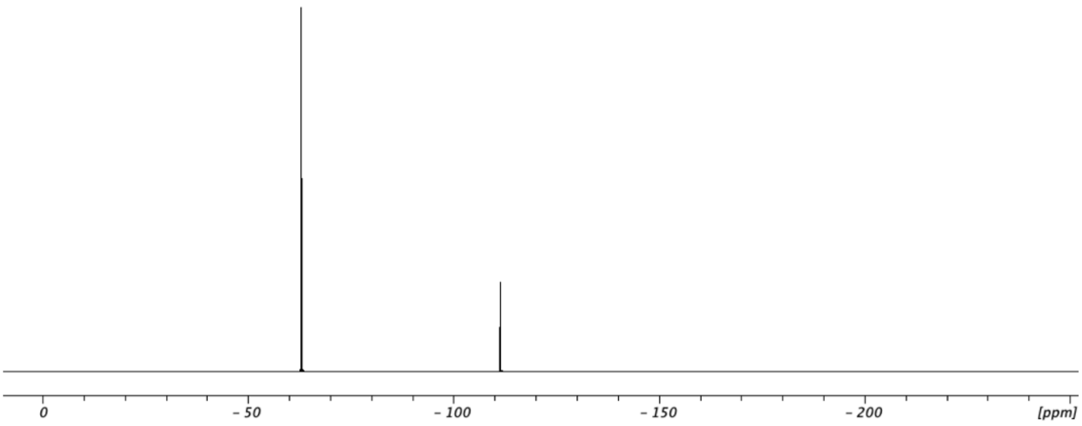
0
- 50
- 100
- 150
- 200
[ppm]

HPLC data

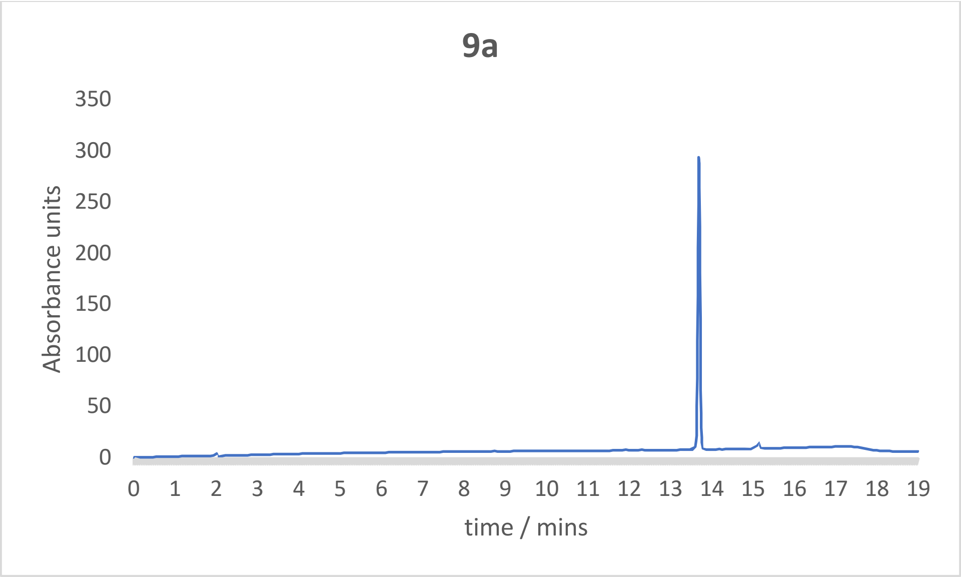
9a
Absorbance units
350
300
250
200
150
100
50
0
0 1 2 3 4 5 6 7 8 9 10 11 12 13 14 15 16 17 18 19
time / mins

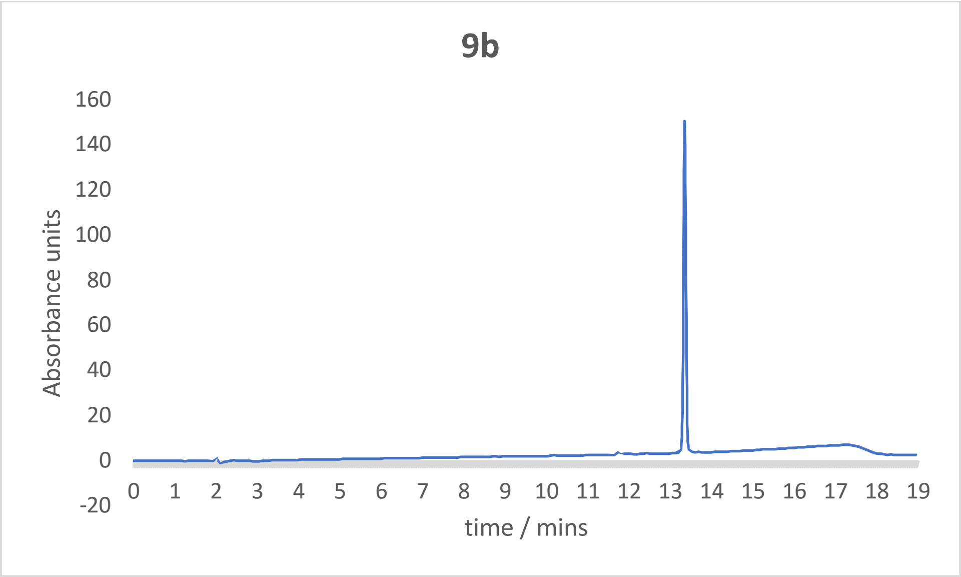
9b
Absorbance units
160
140
120
100
80
60
40
20
0
-20
0 1 2 3 4 5 6 7 8 9 10 11 12 13 14 15 16 17 18 19
time / mins

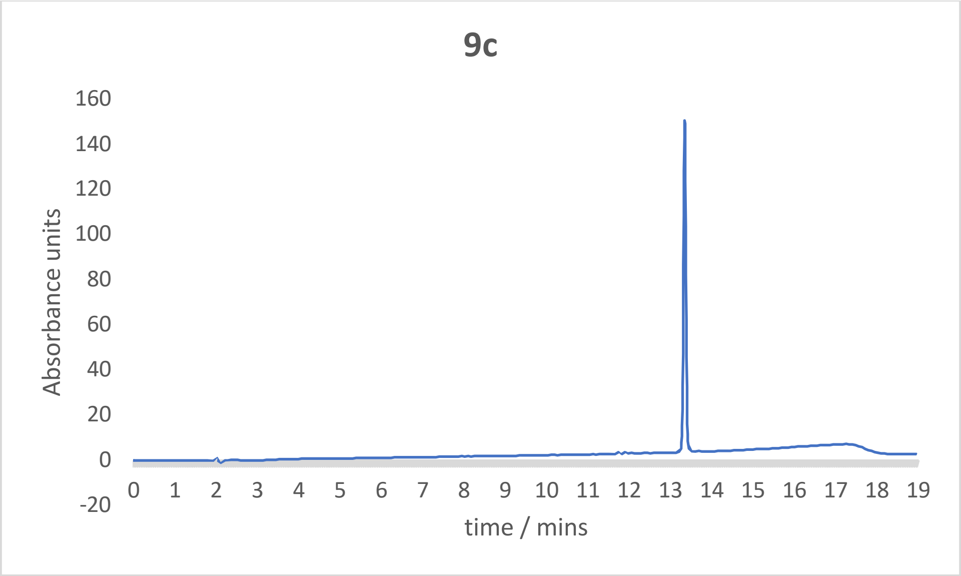
9c
160
140
120
100
80
60
40
20
0
-20
Absorbance units
0 1 2 3 4 5 6 7 8 9 10 11 12 13 14 15 16 17 18 19
time / mins

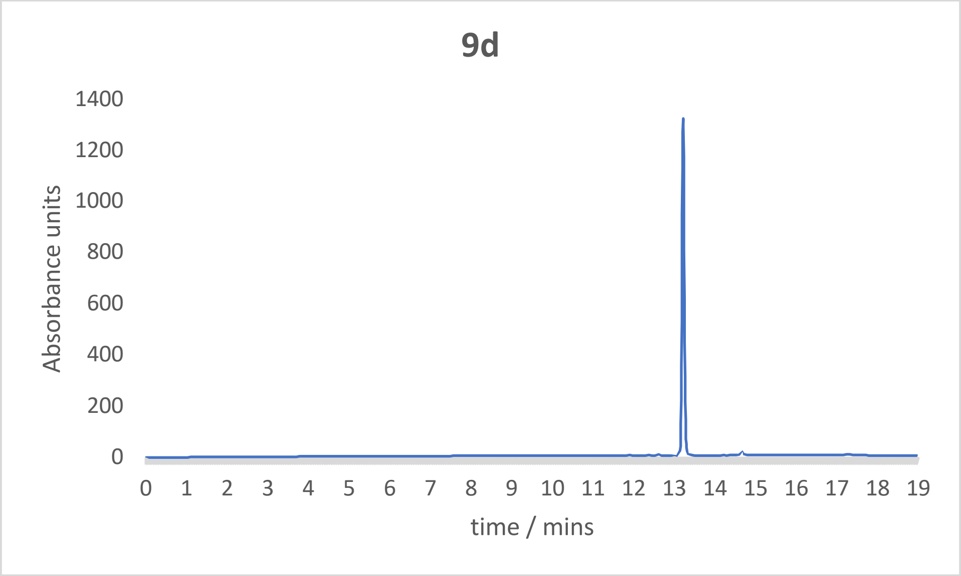
9d
1400
1200
1000
800
600
400
200
0
Absorbance units
0 1 2 3 4 5 6 7 8 9 10 11 12 13 14 15 16 17 18 19
time / mins

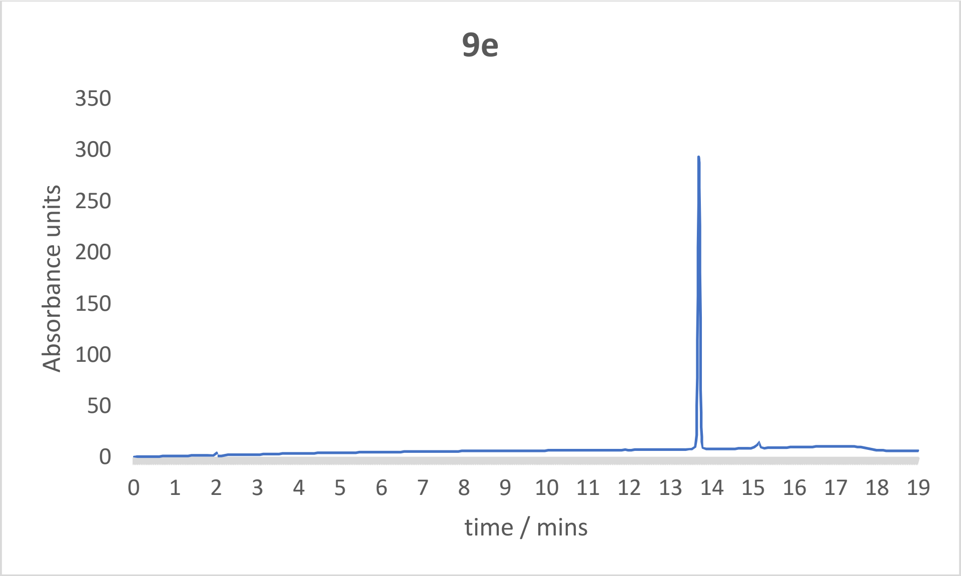
9e
Absorbance units
350
300
250
200
150
100
50
0
0 1 2 3 4 5 6 7 8 9 10 11 12 13 14 15 16 17 18 19
time / mins

Calculated errors for cytotoxicity studies

In order to assess the goodness of fit for the cytotoxicity data, a standard parameter test was applied on the data. Namely, the Sy.X function in GraphPad Prism was used to determine the standard deviation of the residuals (Supplementary table 1).
The Sy.X. function takes into account that the mean of the residuals is always zero, so it roots the sum of squared residuals according to the formula:

$$Sy.x. = \sqrt{\frac{\sum(residual^2)}{n-k}}$$

where k represents the numbers of parameters fit by regression, and n – k represents the number of degrees of freedom for the chosen non-linear regression.

	9a	9b	9c	9d	9e	Enza	EPI	Enza + EPI	Epoxymycin
PC-3	6.9	9.2	7.9	12.3	8.5	18.3	4.6	n.a.	5.4
C4-2b	6	5	5.9	6.7	4.5	6.3	6.1	10.3	n.a.

Supplementary table 1. Standard deviations of the residuals computed for all experiments performed on PC-3 and on C4-2b cells.

Statisitical significance for luciferase assays and RT-qPCR experiments

Unpaired 2-tailed t-test p values (vs. Vehicle control)	9a	9b	9c	9d	9e	Enza	EPI
Figure 3a (luciferase)	<0.0001	0.0083	0.7378	0.0077	<0.0001	<0.0001	0.3803
Figure 3b (RT-qPCR)	0.0419	0.0397	0.7991	0.0612	0.1186	0.0229	0.3232

Supplementary Table 2: p values generated from unpaired two-tailed t-test comparing R1881-induced activation of the AR in Vehicle control cells to cells treated with compounds. Enzalutamide and EPI-001 used as positive controls. p values correspond to figure 3a for luciferase data showing transcriptional activation of the AR and to figure 3b for AR target gene, KLK3 expression when treated with above compounds.
